# An alternatively spliced STING isoform localizes in the cytoplasmic membrane and directly senses extracellular cGAMP

**DOI:** 10.1172/JCI144339

**Published:** 2022-02-01

**Authors:** Xiaobo Li, Yuanyuan Zhu, Xiao Zhang, Xiang An, Mingjiao Weng, Jiaqi Shi, Song Wang, Caiqi Liu, Shengnan Luo, Tongsen Zheng

**Affiliations:** 1 Department of Pathology, Harbin Medical University, Nangang District, Harbin, Heilongjiang, China.; 2 Key Laboratories of Molecular Oncology of Heilongjiang Province, Harbin, Heilongjiang, China.; 3 Department of Gastrointestinal Medical Oncology and; 4 Department of Phase I Trials Center, Harbin Medical University Cancer Hospital, Harbin, Heilongjiang, China.

**Keywords:** Immunology, Oncology, Cancer immunotherapy, Cellular immune response, Innate immunity

## Abstract

It has been revealed that 2′3′-cyclic-GMP-AMP (cGAMP), a second messenger that activates the antiviral stimulator of IFN genes (STING), elicits an antitumoral immune response. Since cGAMP cannot cross the cell membrane, it is not clear how intracellular STING has been activated by extracellular cGAMP until SLC19A1 was identified as an importer to transport extracellular cGAMP into the cytosol. However, SLC19A1-deficient cells also sense extracellular cGAMP, suggesting the presence of mechanisms other than the facilitating transporters for STING sensing extracellular cGAMP. Here, using immunoprecipitation, immunofluorescence, and flow cytometry, we identified an alternatively spliced STING isoform, plasmatic membrane STING (pmSTING), that localized in the plasma membrane with its C-terminus outside the cell, due to a lack of 1 transmembrane domain in its N-terminus compared with canonical STING. Further studies showed that extracellular cGAMP not only promoted the dimerization of pmSTING and interaction of pmSTING with TANK-binding kinase 1 (TBK1) and IFN regulatory factor 3 (IRF3), but also enhanced the phosphorylation of TBK1 and IRF3 and the production of IFN in pmSTING-transfected cells. Additionally, we also identified similar pmSTING isoforms in other species including human. This study suggests a conserved role for pmSTING in sensing extracellular cGAMP and provides insight into the role of cGAMP as an immunotransmitter.

## Graphical abstract

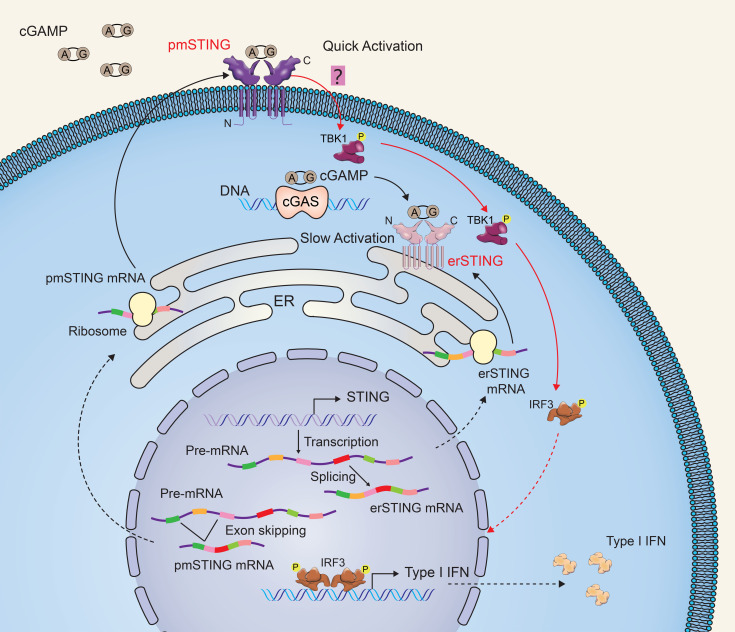

## Introduction

Although it was found as early as 1908 by Mechnikov that DNA can stimulate immune responses, the mechanisms regarding the immune response against DNA by immune cells remained unelucidated throughout the entire 20th century (1). The identification of stimulator of IFN genes (STING), an ER-resident protein containing 4 transmembrane domains, with its C-terminus projecting into the cytoplasm (2), made a great contribution to the field in 2008. And Chen’s group ultimately explained the detailed mechanisms of the DNA-triggered immune responses in 2013 by demonstrating that cyclic GMP-AMP synthase (cGAS) is the direct cytosolic DNA sensor (3, 4). Once it appears in the cytosol upon infection or genomic damage, the cytosolic DNA binds and activates cGAS, an enzyme that causes the synthesis of 2′3′-cyclic-GMP-AMP (cGAMP) by using GMP and AMP as substrates. cGAMP is a second messenger that binds and activates STING (5–8). Upon ligand binding, homodimerized STING translocates from the endoplasmic reticulum to the perinuclear area, where it recruits and activates TANK-binding kinase 1 (TBK1), which phosphorylates and activates IFN regulatory factor 3 (IRF3), a transcription factor that activates the transcription of type I IFNs and other immune mediators (9–11).

The cytosolic DNA–triggered activation of cGAS/STING/IFN signaling not only plays critical roles in the host defense against microbial infection, but is also critical for the antitumor immune response, and numerous studies have suggested that the activation of STING is a promising strategy to treat cancer (1). It has been shown that extracellular cyclic dinucleotides (CDNs) can activate the STING pathway, because the administration of exogenous CDNs has adjuvant effects and antitumor activity in mice (12), suggesting that extracellular CDNs can activate this pathway. However, CDNs cannot pass through the lipid bilayer because of their negative charges. How STING senses extracellular CDNs remains elusive. Facilitating mechanisms that allow CDNs to enter cells have been proposed to explain how STING senses extracellular CDNs. Recently, using a genome-wide CRISPR screen, 2 studies independently identified SLC19A1 as an importer to transport extracellular cGAMP into the cytosol. However, SLC19A1-deficient cells also sense extracellular cGAMP (13, 14), suggesting that mechanisms other than the facilitating transporters for extracellular CDNs that sense STING must be examined. Here, we describe an alternatively spliced STING isoform embedded within the plasma membrane that activates immune responses by direct sensing of extracellular cGAMP.

## Results

### Extracellular cGAMP activates immune responses in a STING-dependent manner.

**Figure 1:**
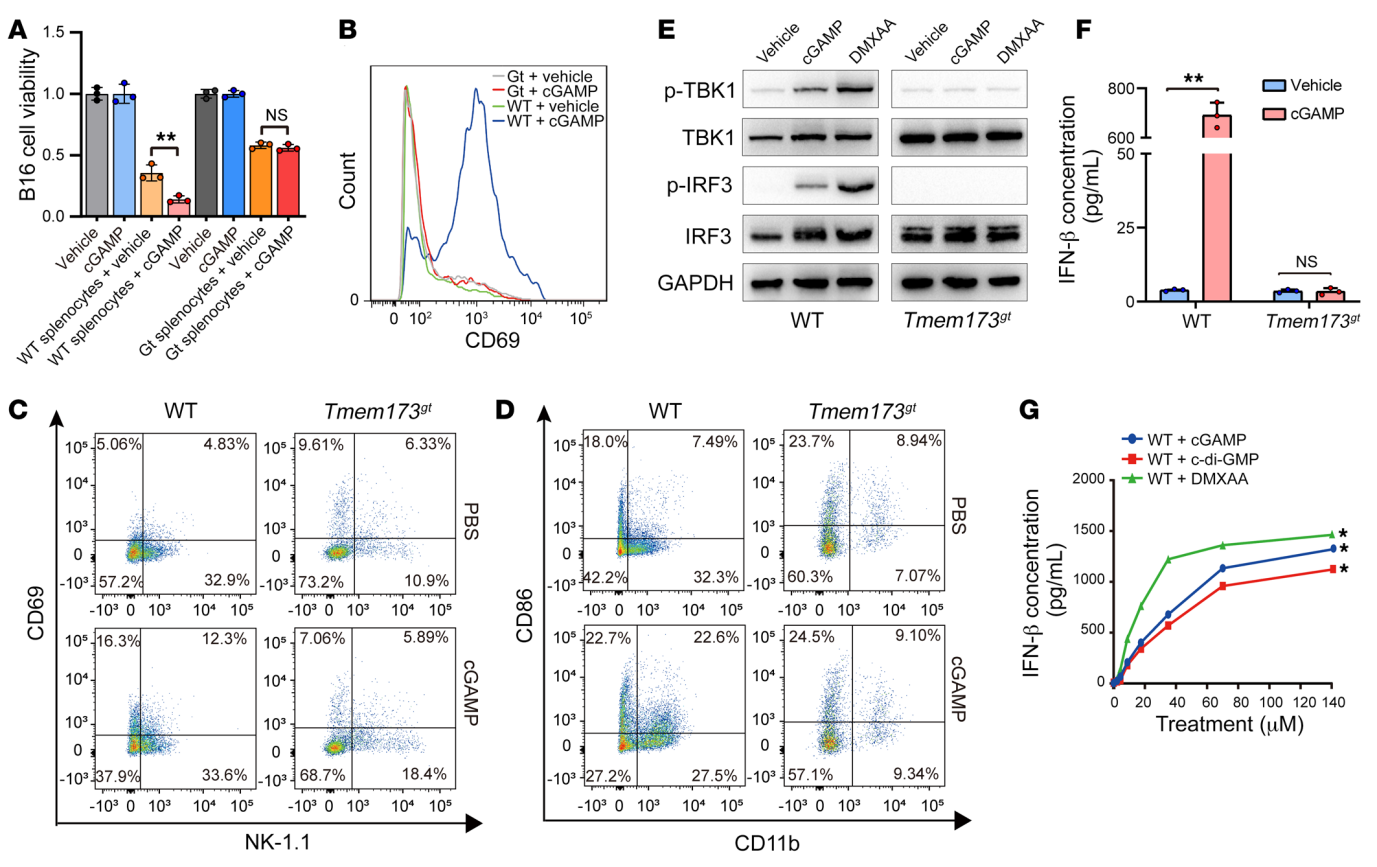
Extracellular cGAMP activates immune responses in a STING-dependent manner. (**A**) The effect of cGAMP on the viability of B16 cells cocultured with or without splenocytes from WT or STING-deficient mice (*Tmem173^gt^*) was assessed by MTT assay (*n =* 3). **P < 0.01, by 2-tailed, paired Student’s *t* test. (**B**) Expression of CD69 in WT or *Tmem173^gt^* splenocytes treated with vehicle or cGAMP was detected by flow cytometry. (**C**) Expression of CD69 in WT or *Tmem173^gt^* NK cells treated with vehicle or cGAMP was detected by flow cytometry. (**D**) Expression of CD86 in WT or *Tmem173^gt^* myeloid cells treated with vehicle or cGAMP was detected by flow cytometry. (**E**) Expression of p-TBK1 and p-IRF3 was detected by Western blotting in WT or *Tmem173^gt^* splenocytes treated with vehicle, cGAMP, or DMXAA, respectively. (**F**) Production of IFN-β in WT or *Tmem173^gt^* splenocytes treated with vehicle or cGAMP was detected by ELISA (*n =* 3). **P < 0.01, by 2-tailed, paired Student’s *t* test. (**G**) Production of IFN-β in WT splenocytes treated with different concentrations of cGAMP, c-di-GMP, or DMXAA was detected by ELISA (*n =* 3). *P < 0.05, by 2-way ANOVA. Data are presented as the mean ± SD.

Although exogenous cGAMP shows antitumor activity in mice, the separate effect of cGAMP on cancer cells and immune cells has not been evaluated simultaneously. We showed that extracellular cGAMP did not affect the viability of B16 melanoma cells, whereas cGAMP promoted the antitumor activity of splenocytes in a STING-dependent manner (Figure 1A), which indicates that the antitumor effect of extracellular cGAMP depends on host immune cells. Then we confirmed that extracellular cGAMP directly activate splenocytes (Figure 1B) and various types of immune cells, such as NK cells (Figure 1C), myeloid cells (Figure 1D), and T cells and B cells (Supplemental Figure 1, A–D; supplemental material available online with this article; https://doi.org/10.1172/JCI144339DS1), in a STING-dependent manner. Moreover, we demonstrate that extracellular cGAMP, similar to the penetrative STING activator DMXAA, activated the STING pathway, as evidenced by an enhanced level of phosphorylation of TBK1 and IRF3 in splenocytes from WT mice but not STING-deficient mice (*Tmem173^gt^*; Figure 1E), and induced the production of IFN-β in a STING-dependent (Figure 1F) and dose-dependent (Figure 1G) manner. These results suggest that extracellular cGAMP directly activates immune responses by stimulating the STING pathway.

### A cell surface STING projecting its C-terminus outside the cell exists in mouse immune cells.

**Figure 2:**
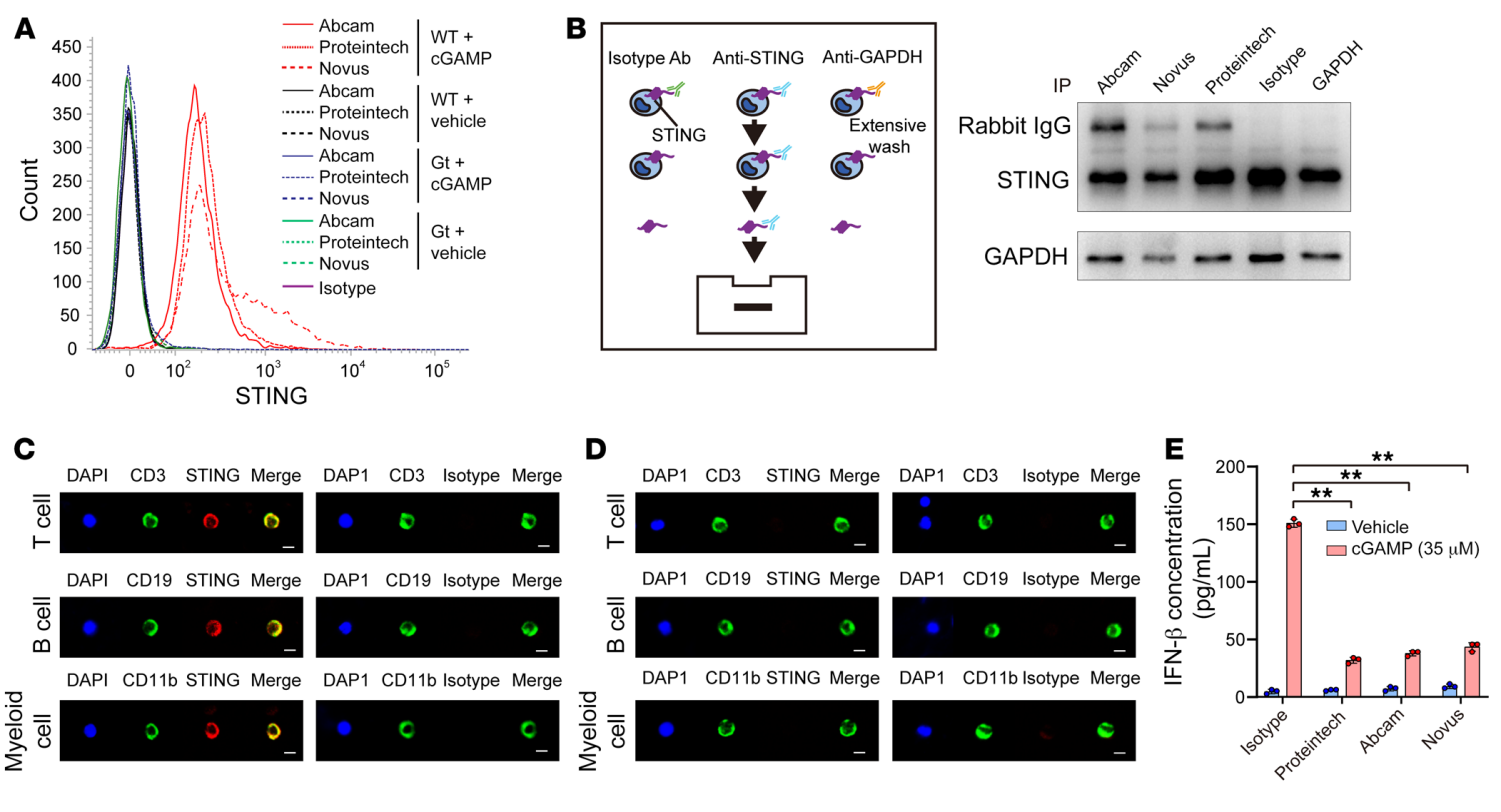
Identification of a cell surface STING projecting its C-terminus outside of splenocytes. (**A**) A cell surface STING with its C-terminus outside of mouse splenocytes was detected with 3 antibodies against the STING C-terminal epitope using flow cytometry. (**B**) WT splenocytes were incubated with the indicated antibodies and then washed and lysed. Immunoblotting was performed to detect the existence of IgG in the cell lysate using a second antibody against rabbit IgG. (**C**) Colocalization of cell surface STING with surface proteins of T cells (CD3), B cells (CD19), and myeloid cells (CD11b) from C57BL/6 mice was detected using confocal microscopy. Scale bars: 5 μm. (**D**) Expression of cell surface STING and surface proteins of T cells (CD3), B cells (CD19), and myeloid cells (CD11b) from STING-deficient mice (*Tmem173^gt^*) using confocal microscopy. Scale bars: 5 μm. (**E**) Extracellular cGAMP–induced production of IFN-β was detected by ELISA in WT splenocytes preincubated with the indicated antibodies (*n =* 3). **P < 0.01, by 1-way ANOVA followed by Dunnett’s test for comparison with the isotype antibody and the cGAMP treatment group.

Recently, SLC19A1 was recognized as being an importer of extracellular cGAMP into the cytosol. However, SLC19A1-deficient cells also sense extracellular cGAMP (13, 14), which suggests that there are unknown mechanisms by which STING senses extracellular cGAMP. Here, the topology of transmembrane protein CD38 may provide us a molecular clue to explain this phenomenon. CD38 is a signaling enzyme that catalyzes the metabolism of cyclic ADP-ribose (cADPR), an intracellular second messenger regulating cellular Ca^2+^ levels. However, its catalytic C-domain localizes outside the cell and binds with extracellular substrates, which induces the internalization of CD38 (15–17). Thus, we sought to determine whether a cell surface STING is located on the plasma membrane with its C-terminus outside the cell, directly sensing cGAMP. Antibodies against the STING C-terminal domain epitope showed immunoreactivity toward nonpermeabilized mouse splenocytes (Figure 2A). We also confirmed the binding of STING antibodies to WT splenocytes by immunoprecipitation (Figure 2B). Moreover, we observed colocalization of STING with surface proteins (CD3, CD19, and CD11b) in WT mice but not in STING-deficient mice (*Tmem173^gt^*) (Figure 2, C and D). Importantly, blocking with antibodies against the STING C-terminus significantly attenuated the production of IFN-β by WT mouse splenocytes in response to extracellular cGAMP (Figure 2E). These results suggest that some STING proteins with their C-terminal cGAMP binding sites outside the cell are expressed on the plasma membrane of mouse immune cells.

### An alternatively spliced mouse STING isoform with 3 transmembrane domains locates in the plasma membrane.

**Figure 3:**
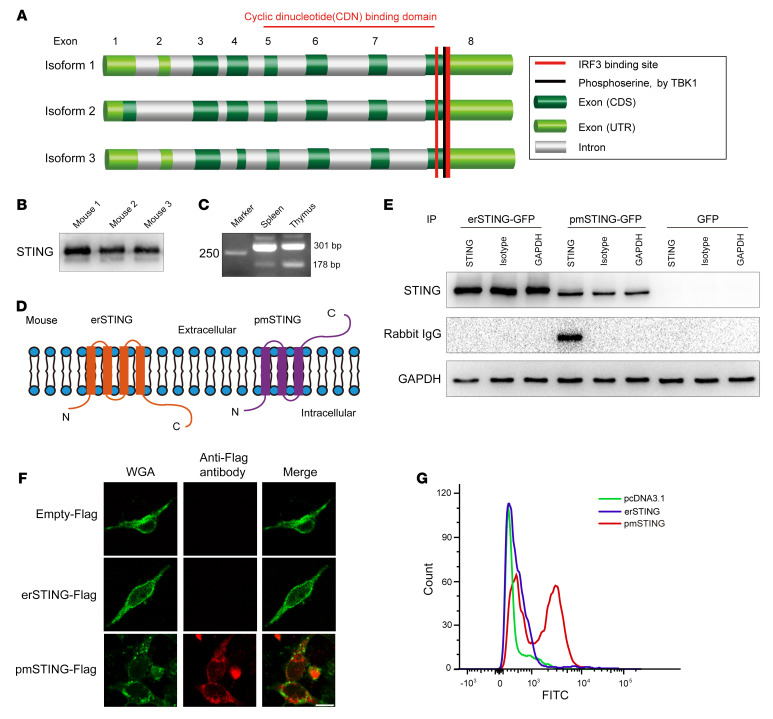
An alternatively spliced STING isoform with 3 TM domains localizes in the plasma membrane of mouse splenocytes. (**A**) Predicted exon structure and schematic of the functional domains in the C-terminus of mouse *Tmem173* transcript variants based on NCBI’s GENE database. (**B**) Different STING isoforms were detected by immunoblotting in splenocytes from 3 individual C57BL/6 mice. (**C**) Two STING isoforms with a different N-terminus were detected by reverse transcription PCR (RT-PCR) in mouse spleen and thymus. (**D**) Predicted plasma membrane topology of detected mouse STING isoforms. (**E**) B16*^Tmem173–/–^* cells transfected with erSTING-EGFP, pmSTING-EGFP, or EGFP were incubated with the indicated antibodies and then washed and lysed. Immunoblotting was performed to detect IgG in the cell lysate using a secondary antibody against rabbit IgG. (**F** and **G**) B16*^Tmem173–/–^* cells were transfected with erSTING-Flag, pmSTING-Flag, or a vector plasmid, respectively. An antibody against Flag was used to detect Flag projecting outside cells using immunofluorescence (**F**) and flow cytometry (**G**), respectively. Scale bars: 20 μm.

In mice, the *Tmem173* gene is predicted to encode 3 alternatively spliced STING isoforms with an identical C-terminus but a different N-terminus, based on the NCBI’s GENE database (gene ID: 72512; Figure 3A). Thus, we used an antibody against the C-terminus of STING to determine whether all of these predicted STING isoforms were expressed in mice splenocytes, and an additional isoform shorter than the canonical STING was identified (Figure 3B). Additionally, 2 alternatively spliced STING isoforms were also detected by RT-PCR (Figure 3C) and validated by sequencing (Supplemental Figure 2A) in splenocytes. We also found that both STING isoforms were ubiquitously expressed in different mouse tissues (Supplemental Figure 2, B and C). Moreover, the C-terminus of the shorter spliced STING isoform (plasmatic membrane STING, pmSTING) was predicted to be outside the cell because of the lack of a transmembrane (TM) domain compared with the canonical isoform endoplasmic reticulum STING (erSTING) (Figure 3D). To investigate the topology of pmSTING and erSTING, we constructed a pmSTING-GFP (or pmSTING-Flag) and an erSTING-GFP (or erSTING-Flag) fusion protein and then expressed them in B16*^Tmem173–/–^* cells. We demonstrated that the C-terminus of pmSTING indeed faced outside the cell (Figure 3E), using immunoprecipitation, immunofluorescence (Figure 3F), and flow cytometry (Figure 3G).

### The mouse pmSTING isoform directly senses extracellular cGAMP and activates TBK1/IRF3/IFN signaling.

**Figure 4:**
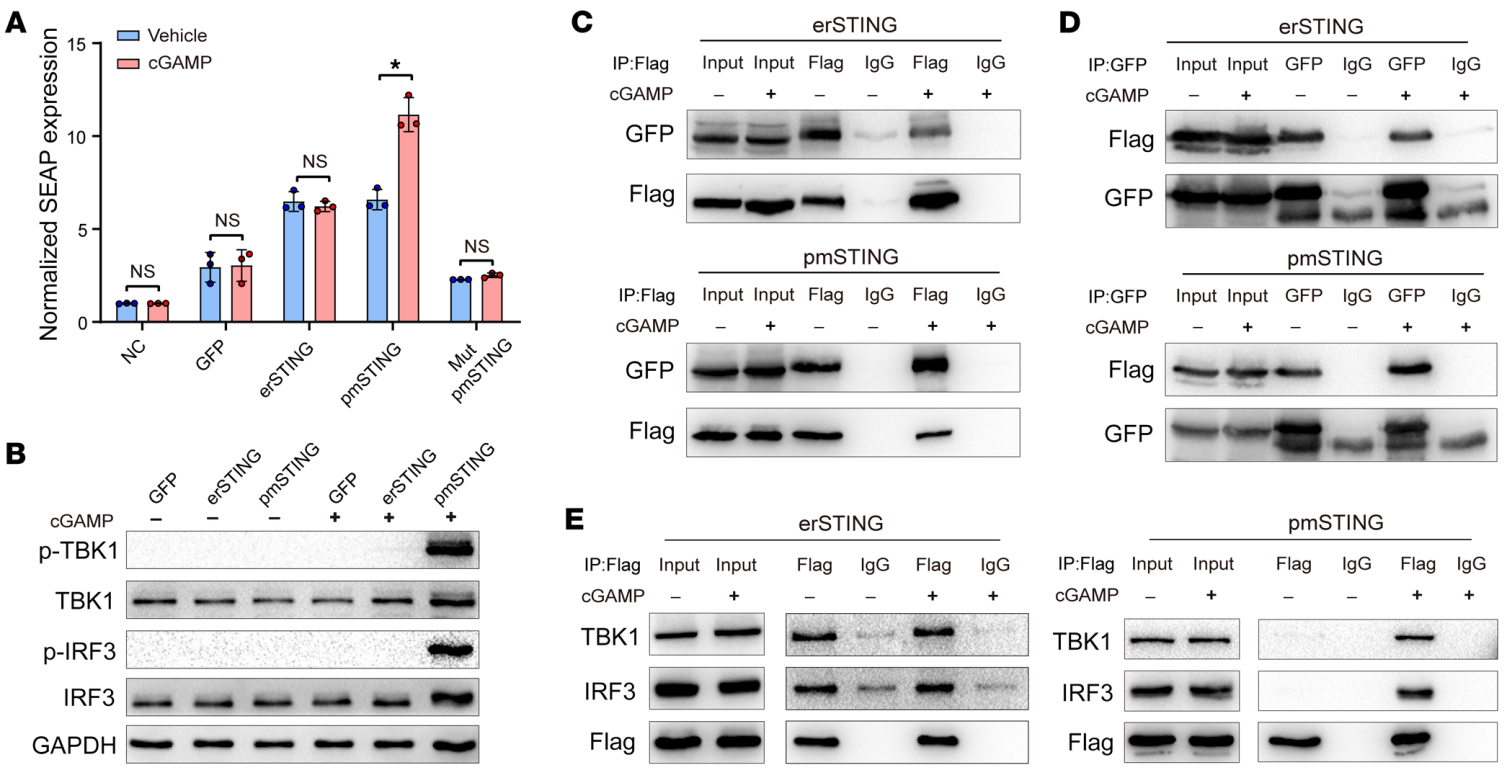
Mouse pmSTING isoform directly senses extracellular cGAMP and activates TBK1/IRF3/IFN signaling. (**A**) B16*^Tmem173–/–^* cells stably expressing SEAP to monitor IFN activity were transfected with erSTING-Flag, pmSTING-Flag, or mutated pmSTING-Flag (Mut pmSTING-Flag) at the TBK1 phosphorylation site (Ser316R). SEAP was detected in these cells after treatment with vehicle or cGAMP (*n =* 3). *P < 0.05, by 2-tailed, paired Student’s *t* test. (**B**) Western blotting was performed to detect p-TBK1 and p-IRF3 in B16*^Tmem173–/–^* cells transfected with erSTING-Flag or pmSTING-Flag upon treatment with vehicle or cGAMP. (**C** and **D**) B16*^Tmem173–/–^* cells were transfected with both erSTING-Flag and erSTING-EGFP or both pmSTING-Flag and pmSTING-EGFP, and then treated with vehicle or cGAMP. Immunoprecipitation using anti-Flag antibody (**C**) or anti-GFP antibody (**D**) was performed to detect pmSTING or erSTING dimerization in response to extracellular cGAMP. (**E**) B16*^Tmem173–/–^* cells were transfected with erSTING-Flag or pmSTING-Flag and then treated with vehicle or cGAMP. Immunoprecipitation using anti-Flag antibody was performed to detect the interaction between pmSTING (or erSTING) and TBK1 or IRF3, respectively.

Next, we evaluated the roles of mouse pmSTING in sensing extracellular cGAMP and activating TBK1/IRF3/IFN signaling. A functional study showed that the pmSTING rather than the erSTING isoform specifically sensed extracellular cGAMP and induced the production of IFN in B16*^Tmem173–/–^* cells that stably expressed secreted alkaline phosphatase (SEAP) to monitor IFN activity. However, mutation of the phosphorylation site of TBK1 (S316R) in the C-terminus of pmSTING eliminated its capability to induce SEAP production (Figure 4A). Unexpectedly, B16*^Tmem173–/–^* cells transfected with erSTING or pmSTING showed obviously elevated SEAP levels compared with levels in untransfected cells (Figure 4A). We suspected that endogenous cGAMP due to plasmid DNA–triggered activation of cGAS may contribute to the relatively high basal levels of SEAP in B16*^Tmem173–/–^* cells transfected with erSTING or pmSTING. As expected, we confirmed that knockdown of cGAS decreased the basal levels of SEAP and did not affect pmSTING sensing of extracellular cGAMP (Supplemental Figure 3, A and B). Moreover, we detected the potential roles of SLC19A1 in pmSTING sensing of extracellular cGAMP and showed that SLC19A1 was not necessary for the activation of pmSTING induced by extracellular cGAMP (Supplemental Figure 3, C and D). Additionally, we showed that extracellular cGAMP enhanced the phosphorylation of TBK1 and IRF3 (Figure 4B), induced the dimerization of pmSTING (Figure 4, C and D), and promoted the interaction of pmSTING with TBK1 and IRF3 (Figure 4E) in pmSTING-transfected B16*^Tmem173–/–^* cells, but we did not observe these results in erSTING-transfected B16*^Tmem173–/–^* cells (Figure 4, C–E). Collectively, these results suggest that the pmSTING isoform is expressed in mice at the cell surface with its C-terminus outside the cell and directly senses extracellular cGAMP.

### An alternatively spliced isoform of human STING with 1 transmembrane domain embeds in the plasma membrane and projects its C-terminus outside the cell.

**Figure 5:**
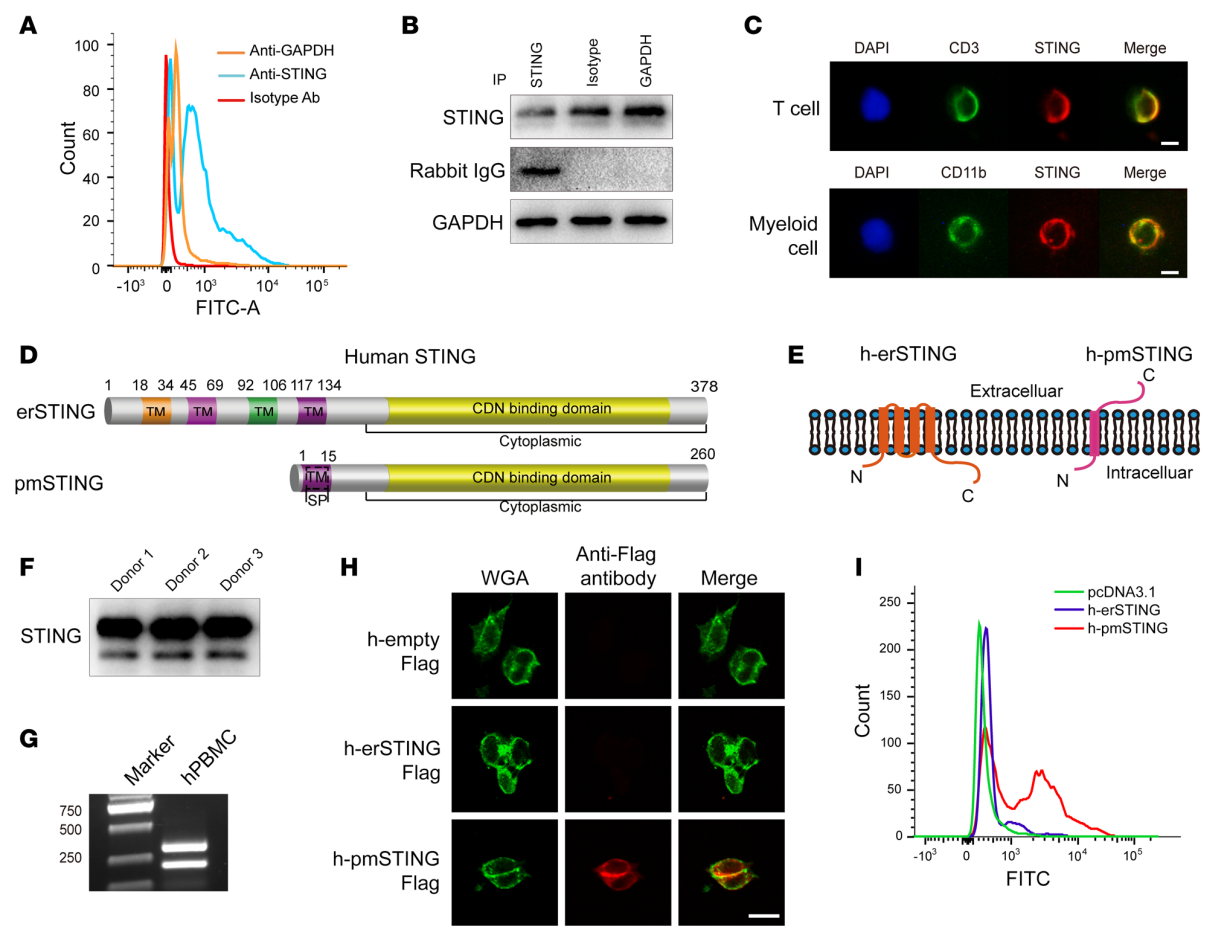
An alternatively spliced isoform of human STING localizes in the plasma membrane. (**A**) Flow cytometry was performed to detect cell surface STING with its C-terminus outside human PBMCs. (**B**) Human PBMCs were incubated with the indicated antibodies and then washed and lysed. Immunoblotting was performed to detect IgG in the cell lysate using a secondary antibody against rabbit IgG. (**C**) Colocalization of cell surface STING with surface protein of T cells (CD3) and myeloid cells (CD11b) from human PBMCs was detected by confocal microscopy. Scale bars: 10 μm. (**D**) Exon structure of the predicted human *TMEM173* transcript variants based on the NCBI’s GENE database. (**E**) Predicted plasma membrane topology of STING isoforms in homo sapiens. (**F**) Two STING isoforms were detected by an antibody against the STING C-terminal epitope using immunoblotting of human PBMCs from 3 healthy donors. (**G**) Two STING isoforms with a different N-terminus were detected in human PBMCs (hPBMC) by RT-PCR. (**H** and **I**) 293T cells were transfected with h-erSTING-Flag, h-pmSTING-Flag, or a vector plasmid, respectively. An antibody against Flag was used to detect Flag projecting outside of cells by immunofluorescence (**H**) and flow cytometry (**I**), respectively. Scale bar: 20 μm.

Identification of a pmSTING in mice led us to consider if a pmSTING isoform also existed in humans. We confirmed that a pmSTING with C-terminus outside cells exists in human PBMCs using flow cytometry (Figure 5A), immune precipitation (Figure 5B), and immunofluorescence (Figure 5C) assays. In addition to encoding the canonical STING isoform (human erSTING [h-erSTING]), the human *TMEM173* gene was also predicted to encode an alternative spliced STING isoform with 1 TM domain based on the NCBI’s Gene database (gene ID: 340061; Figure 5, D and E), and the expression of this transcript was confirmed in PBMCs by immunoblotting (Figure 5F) as well as by PCR and sequencing (Figure 5G and Supplemental Figure 4). We further demonstrated that this human pmSTING (h-pmSTING) was expressed in the plasma membrane and that its C-terminus was localized in the extracellular space using immunofluorescence (Figure 5H) and flow cytometry (Figure 5I).

### h-pmSTING directly senses extracellular cGAMP and activates TBK1/IRF3/IFN signaling.

**Figure 6:**
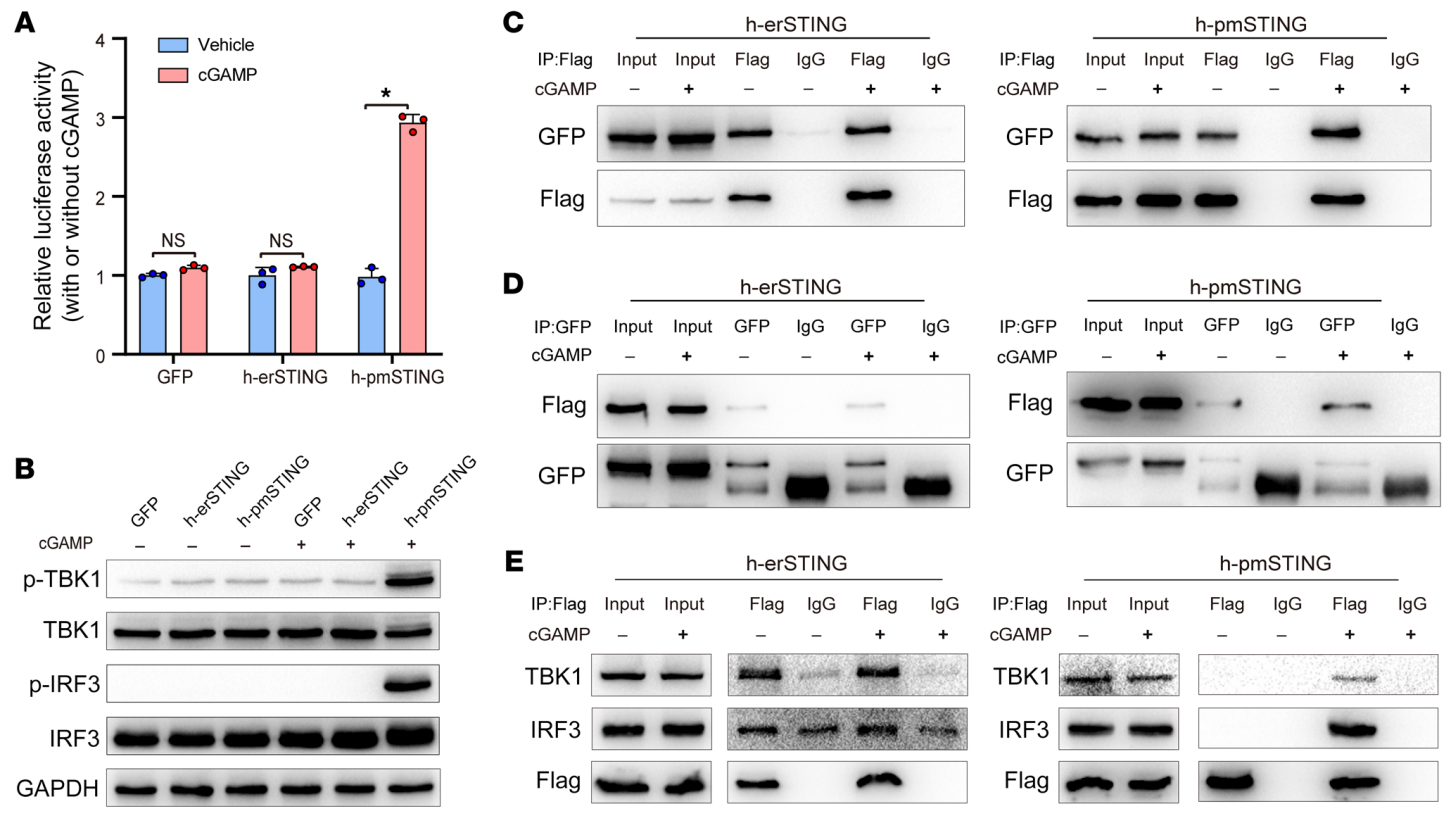
Human pmSTING isoform also directly senses extracellular cGAMP and activates TBK1/IRF3/IFN signaling. (**A**) 293T cells were cotransfected with h-erSTING-Flag or h-pmSTING-Flag and a luciferase reporter to detect IFN production. Luciferase activity was detected in these cells after treatment with vehicle or cGAMP (*n =* 3). *P < 0.05, by 2-tailed, paired Student’s *t* test. (**B**) Western blotting was performed to detect p-TBK1 and p-IRF3 levels in 293T cells transfected with h-erSTING-Flag or h-pmSTING-Flag upon treatment with vehicle or cGAMP. (**C** and **D**) 293T cells were transfected with both h-erSTING-Flag and h-erSTING-EGFP or both h-pmSTING-Flag and h-pmSTING-EGFP, and then treated with vehicle or cGAMP. Immunoprecipitation using anti-Flag antibody (**C**) or anti-GFP antibody (**D**) was performed to detect the dimerization of h-pmSTING or h-erSTING in response to extracellular cGAMP. (**E**) 293T cells were transfected with h-erST1 ING-Flag or h-pmSTING-Flag and then treated with vehicle or cGAMP. Immunoprecipitation using anti-Flag antibody was performed to detect the interaction between h-pmSTING (or h-erSTING) and TBK1 or IRF3, respectively.

Furthermore, we investigated whether such a h-pmSTING isoform also contributes to sensing of extracellular cGAMP and activation of TBK1/IRF3/IFN signaling. We showed that extracellular cGAMP not only induced IFN production (Figure 6A) and enhanced the phosphorylation of TBK1 and IRF3 (Figure 6B) in h-pmSTING–transfected rather than h-erSTING–transfected 293T cells, but also promoted the dimerization of h-pmSTING (Figure 6, C and D) and interaction of h-pmSTING with TBK1 and IRF3 (Figure 6E) in h-pmSTING–transfected 293T cells. By comparison, extracellular cGAMP had no effect on the dimerization of h-erSTING (Figure 6, C and D) or the interaction of h-erSTING with TBK1 and IRF3 (Figure 6E) in h-erSTING–transfected 293T cells. These results suggest that pmSTING, rather than erSTING, directly sensed extracellular cGAMP and activated TBK1/IRF3/IFN signaling in human cells.

**Figure 7:**
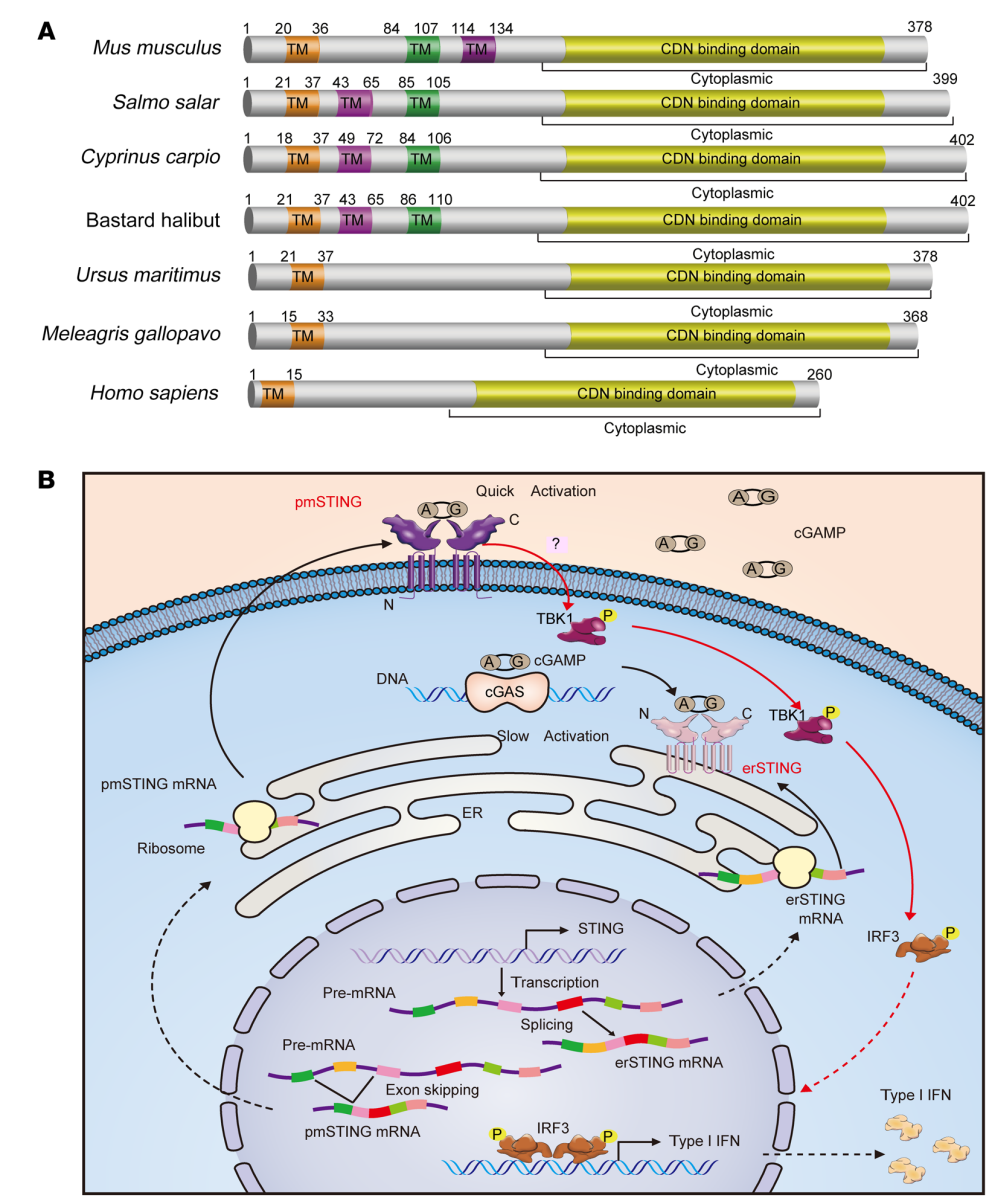
Schematic of the predicted pmSTING in various species and model of pmSTING sensing extracellular cGAMP and prompting IFN production. (**A**) Multiple protein sequence alignment of STING proteins orthologous to the mouse pmSTING isoform. The STING proteins in different species shown here meet the following criteria: (a) possess an odd number of predicted TM domains; and (b) possess a C-terminal domain identical to that of the respective species’ canonical STING protein. (**B**) Model of the generation of 2 alternatively spliced STING isoforms and how they sense extracellular and intracellular cGAMP, respectively. Two STING transcripts generated by alternative splicing are translated in the cytoplasm and transported to the pmSTING and the endoplasmic reticulum (erSTING), respectively. Upon binding the intracellular cGAMP synthesized by cytosolic DNA–activated cGAS, erSTING undergoes homodimerization and translocates from endoplasmic reticulum to the perinuclear area, where it recruits and activates TBK1, which phosphorylates the transcription factor IRF3 and results in the translocation of IRF3 from the cytoplasm to the nucleus to induce the transcription of IFN and other immune cytokines. By contrast, the extracellular cGAMP released by dead cells directly binds pmSTING and causes homodimerization and translocation of pmSTING from the plasmatic membrane to the perinuclear area, where it activates TBK1/IRF3/IFN signaling.

Finally, we evaluated whether such a pmSTING isoform with its C-terminus outside the cell is conserved across animals. On the basis of the alternatively spliced isoform sequence, we predicted that the STING isoform with its C-terminus outside the cell also exists in many other species (Figure 7A), and that pmSTING may be a conserved membrane molecule sensing extracellular cGAMP in the animal kingdom.

## Discussion

The canonical STING resides in the endoplasmic reticulum, thus activation of STING requires cytoplasmic localization of its ligands (3, 18, 19). This has inspired several strategies to deliver the CDNs into the cytoplasm either through cell permeabilization or liposome-mediated transfection (20–23). Recently, it was reported that some membrane molecules facilitate transfers of extracellular CDNs into the cytosol. For example, upon infection, cGAMP could be transferred from HSV-1–infected cells into bystander cells through LRRC8, a subunit of volume-regulated anion channels (24). In cancer tissues, cGAMP released from dying cells could enter tumor-associated macrophages and activate STING signaling through the ATP-gated channel P2X7R (25). However, it is unknown if LRRC8 and P2X7R facilitate transfers of extracellular cGAMP in other settings. Two research groups independently determined that the reduced folate carrier SLC19A1 is a direct cGAMP importer in many cell types. However, SLC19A1-deficient cells also sense extracellular cGAMP (13, 14). Consistently, we showed that knockdown of SLC19A1 expression in B16*^Tmem173–/–^* cells did not affect the ability of pmSTING to sense extracellular cGAMP (Supplemental Figure 3, C and D). Collectively, these results suggest that facilitating transporters may not be the only mechanism for the sending of extracellular CDNs by STING.

We identified an alternatively spliced STING isoform that ubiquitously presents in the plasma membrane of human and mouse cells, with its C-terminus extending to the outside of the cell surface. More interestingly, we also identified a similar STING isoform in other animal species in our study, suggesting a broad and conserved role for pmSTING in the sensing of extracellular CDNs. Additionally, it was found that the canonical STING ectopically expressed in STING-deficient cells could only be activated by intracellular, but not extracellular, cGAMP (26). Consistently, we showed that pmSTING, rather than erSTING, could be activated by extracellular cGAMP in both human and mouse cells, supporting the idea that the pmSTING isoform is the sensor for extracellular cGAMP.

The C-terminus of STING provides the cGAMP binding domain and signaling transduction domains. We showed that pmSTING shared the same C-terminus with the canonical STING but projected its C-terminus outside cells. This membrane topology seems paradoxical to the notion of pmSTING mediation of the type I IFN response. A similar phenomenon has been observed in CD38, a single transmembrane protein with its catalytic C-domain outside the cell. CD38 can undergo an extensive internalization upon binding extracellular ligands (15–17). Although the mechanism underlying this ligand-induced internalization has not been fully explained, internalization of cell surface CD38 results in a shift of exocellular cADPR metabolism to the cytosol (27). In this study, we showed the essential roles of the C-terminus in pmSTING sensing of extracellular cGAMP and observed the internalization of pmSTING upon stimulation by extracellular cGAMP (Supplemental Figure 5, A and B). However, the molecular mechanism by which extracellular cGAMP activates pmSTING translocation remains unclear. The plasma membrane protein Tspan8, which contains 4 transmembrane domains, was found to translocate from the plasma membrane to the nucleus. Mechanistically, it is not in the form of vesicles, but rather a Tspan8-cholesterol complex to be translocated into nuclei, and cholesterol is critical for binding and protecting the hydrophobic transmembrane domains of Tspan8 during the translocation process (28), which suggests another mechanism of plasma membrane protein internalization mediated by a nonspecific transporter, such as cholesterol. Moreover, the canonical STING translocates from the endoplasmic reticulum membrane to the perinuclear area to activate TBK1 and IRF3 upon binding cGAMP, and the translocator in the endoplasmic reticulum composed of TRAPβ, Sec61β, and Sec5 (11, 29) contributes to the translocation of STING, whereas iRhom2 facilitates the assembly of the STING-TRAPβ translocation complex (30). Thus, unknown vector complexes or transporters may contribute to the internalization of pmSTING. However, the detailed molecular mechanisms of pmSTING internalization triggered by extracellular cGAMP need to be further investigated.

In conclusion, we found that an alternatively spliced STING isoform embedded within the plasma membrane with its C-terminus outside the cell directly sensed extracellular cGAMP and activated TBK1/IRF3/IFN signaling (Figure 7B). This study provides insight into the role of cGAMP as an immunotransmitter and may contribute to the development of useful cancer therapeutics targeting cell surface STING.

## Methods

### Cell culturing.

The HEK293T cell line was obtained from the Cell Culture Center of the Chinese Academy of Medical Science and cultured in DMEM (Gibco, Thermo Fisher Scientific) supplemented with 10% FBS (Gibco, Thermo Fisher Scientific) and 0.1% penicillin-streptomycin solution (Biosharp). The B16-Blue ISG-KO-STING cell line (InvivoGen, Thermo Fisher Scientific), the B16 cell line (Cell Culture Center of the Chinese Academy of Medical Science), mouse splenocytes, and human PBMCs were cultured in RPMI 1640 (Gibco, Thermo Fisher Scientific) and a 10% FBS and 0.1% penicillin-streptomycin solution. All cells were maintained in standard culture conditions (37°C in 5% CO_2_).

### Animal models.

C57BL/6J WT mice were purchased from the animal center of the Second Affiliated Hospital of Harbin Medical University. STING-deficient mice (*Tmem173^gt^*) generated by a forward genetic mutagenesis screen in C57BL/6J mice using the mutagen *N*-ethyl-*N*-nitrosourea were purchased from The Jackson Laboratory (31). All mice were maintained in specific pathogen–free conditions with ad libitum access to food and water.

### Isolation of splenocytes.

Fresh spleens were harvested from WT and STING-deficient mice (*Tmem173^gt^*) and gently crushed by the inner piston of the syringe in sterile PBS (Solarbio). Next, splenocytes were suspended in PBS and filtrated with a 100 μm mesh filter and concentrated at 1500*g*. The cell precipitation was resuspended in 1 mL RBC lysis buffer to remove the RBCs for 2 minutes. The cell precipitation was resuspended in PBS after concentration 2 times at 1500*g*.

### Isolation of human PBMCs.

Blood from healthy volunteers was collected into 5 mL EDTA anticoagulation tubes and diluted with 6 mL sterile PBS. Then, the diluted blood was gently added to the tube containing 5 mL Ficoll-Hypaque (TBDsciences) solution on the bottom. The tube was centrifuged at 1500*g*. After centrifugation, the solution in the tube was divided into 3 layers, and the intermediate froggy layer containing PBMCs was transferred into a new tube and underwent another centrifugation at 1500*g*. The cell precipitation was resuspended in 1 mL RBC lysis buffer to remove the RBCs for 2 minutes. After centrifugation at 1500*g*, the cell precipitation was resuspended in PBS.

### Flow cytometry.

Mouse splenocytes were stimulated with 35 μM 2′,3′-cGAMP (InvivoGen) or 35 μM DMXAA (Selleck Chemicals) for 18 hours. Human PBMCs were stimulated with 35 μM 2′,3′-cGAMP for 18 hours. Cells were collected and centrifuged at 1500*g* after stimulation by cGAMP or DMXAA. Then, cell precipitations were washed twice in PBS. After adjusting the cell number to 2 × 10^6^, surface staining was performed with the following fluorochrome-conjugated antibodies at 4°C in the dark for 30 minutes: FITC anti–mouse CD3 antibody (17A2), PE anti–mouse CD69 antibody (H1.2F3), FITC anti–human CD3 antibody (HIT3a), PE anti–human CD69 antibody (FN50), FITC anti–mouse CD4 antibody (GK1.5), APC anti-mouse CD8 antibody (CD8 53-6.7), FITC anti–mouse CD19 antibody (6D5), PE anti–mouse CD86 antibody (GL-1), FITC anti–mouse CD11b antibody (M1/70), and PE/cyanine7 anti–mouse NK-1.1 antibody (PK136) were all purchased from BioLegend. To identify the plasma membrane STING isoform, cells were incubated with the primary antibodies against the C-terminus of STING (19851-1-AP, Proteintech; ab92605, Abcam; NBP2-24683SS, Novus Biologicals), anti-GAPDH antibody (60004-1-Ig, Proteintech) and rabbit IgG isotype antibody (MilliporeSigma) at 4°C for 1 hour. B16-KO-STING cells transfected with erSTING-Flag or pmSTING-Flag were stimulated with 35 μM 2′,3′-cGAMP for 18 hours, and cells were incubated with anti-Flag antibody (14793S, Cell Signaling Technology) at 4°C for 1 hour. After washing twice in PBS, cells were stained by the FITC-conjugated secondary antibody (SA00003-2, Proteintech) at room temperature in the dark for 1 hour.

After staining, cells were washed twice in PBS (1500*g*, 5 minutes) and resuspended with 500 μL PBS and filtrated through a mesh filter (50 μm). All samples were examined by BD LSR Fortessa and analyzed with FlowJo software (TreeStar).

### In vitro killing assay.

Melanoma B16 cells were seeded in the 96-well plate at 2000 cells per well and were cocultured with or without splenocytes (the ratio of splenocytes and B16 cells was 50:1) and treated with 35 μM cGAMP or vehicle control for 48 hours. Twenty microliters of 0.5% MTT solution (Solarbio) was added to each well of the plate and incubated at 37°C in the dark for 4 hours. The supernatant was gently replaced with 150 μL DMSO, and the plate was shaken for 10 minutes. Cell viability was reflected by the absorption at 490 nm detected by a Spectra Max M5 Multi-Mode Microplate Reader.

### ELISA.

Mouse splenocytes freshly isolated from 2-month-old WT or STING-deficient mice (*Tmem173^gt^*) were incubated in a 96-well plate at a 2 × 10^6^/cells well. After stimulation with 2′,3′-cGAMP, c-di-GMP (InvivoGen), or DMXAA for 24 hours, the splenocyte supernatants were collected. In the STING C-terminus blocking assay, the mouse splenocytes were incubated separately with 10 μg/mL of different antibodies against the C-terminus of STING (19851-1-AP, Proteintech; ab92605, Abcam; NBP2-24683SS, Novus Biologicals) at 37°C for 2 hours before stimulation with cGAMP for 24 hours. The IFN-β in the supernatants of splenocytes was detected with the mouse IFN-β ELISA Kit (42400-1, R&D Systems), following the manufacturer’s protocols.

### Western blot analysis.

Total proteins were extracted from the collected cell pellets using RIPA buffer (Beyotime Institute of Biotechnology), with the addition of the protease inhibitor PMSF (Roche) and phosphatase inhibitors (Roche). Proteins were quantified using a BCA Kit (Beyotime Institute of Biotechnology). Equal protein was separated on an SDS-PAGE gel by electrophoresis and transferred onto a PVDF membrane (MilliporeSigma). After blocking with 5% BSA at room temperature for 1 hour, membranes were incubated with the following primary antibodies: anti–STING rabbit polyclonal antibody (19851-1-AP, Proteintech) diluted at 1:600, anti–mouse TBK1 antibody (ab40676, Abcam) diluted at 1:1000; anti–mouse phosphorylated TBK1 (p-TBK1) (Ser172) antibody (ab109272, Abcam) diluted at 1:500; anti–mouse IRF3 rabbit polyclonal antibody (11312-1-AP, Proteintech) diluted at 1:1000; anti–mouse p–IRF-3 (Ser396) rabbit mAb (29047, Cell Signaling Technology) diluted at 1:1000; anti–mouse cGAS antibody (ab252416, Abcam) diluted at 1:1000; anti–mouse SLC19A1 antibody (25958-1-AP, Proteintech) diluted at 1:1000; and anti–GAPDH mouse mAb (60004-1-Ig, Proteintech) diluted at 1:1000. After washing, the membranes were incubated with a peroxidase-conjugated secondary antibody (SA00001-1 and SA00001-2, Proteintech, diluted at 1:2000) for 1 hour at 37°C. The ECL system and Bio-RAD Gel Doc XR+ system were used to visualize the blots. All assays were replicated 3 times. See the complete unedited blots in the supplemental material.

### Immunoprecipitation.

Mouse splenocytes or human PBMCs were incubated with 5 μg/mL primary antibodies (rabbit anti-STING antibody (ab92605, Abcam; 19851-1-AP, Proteintech; NBP2-24683, Novus Biologicals); rabbit anti-GAPDH antibody (60004-1-Ig, Proteintech); and rabbit IgG isotype antibody (B900610, Proteintech) at 4°C for 1 hour. The cell pellets were collected by centrifugation at 6000*g* for 5 minutes and then lysed with RIPA buffer with PMSF on ice for 10 minutes. The HRP-conjugated goat anti–rabbit IgG antibody (SA00001-2, Proteintech) (1:2000) was used to detect binding of rabbit IgG antibodies on the cell membrane.

### Coimmunoprecipitation.

B16-Blue ISG-KO-STING cells were cotransfected with pmSTING-Flag and pmSTING-GFP or with erSTING-Flag and erSTING-GFP using jetPRIME transfection reagent (Polyplus Transfection) according to the manufacturer’s instructions. Twenty-four hours after transfection, cells were treated with or without cGAMP (35 μM) for 4 hours and then harvested and lysed on ice using prechilled immunoprecipitation lysis buffer with 1× protease inhibitor for 30 minutes. The supernatant was collected by centrifugation at 4°C and 10,000*g* for 20 minutes. Anti-Flag antibody (4 μg, T0003, Affinity) or anti-GFP antibody (50430-2-AP, Proteintech) or the same amount of IgG was added to 500 μL lysate containing 3 mg total protein in spin columns and incubated overnight at 4°C. Next, 50 μL resuspended protein A sepharose beads slurry was added to the spin columns and incubated at 4°C for 4 hours to precipitate the immune complex. The supernatant was removed naturally, and the precipitated complex was washed 5 times with 800 μL 1× washing buffer (containing 1× protease inhibitor). The spin columns were placed into a new 1.5 mL EP tube, and the precipitated complex was eluted with 40 μL elution buffer by centrifugation at 10,000*g* for 1 minute. Finally, 10 μL alkali neutralization buffer and 5× sample buffer were added to the eluted solutions. The immunoprecipitated GFP or Flag was detected by Western blotting.

### PCR.

Total RNA was extracted with TRIzol Reagent (Invitrogen, Thermo Fisher Scientific) according to the manufacturer’s instructions. Total RNA (1 μg) was used to synthesize cDNA using Reverse Transcriptase M-MLV (RNase H, H2640A, Takara) following the manufacturer’s protocol.

The PCR reaction mixture containing 1 μL cDNA, 10 μL 2× Pfu Mix (Sciencestar), 2 μL primers, and 7 μL nuclease-free water underwent 35 PCR cycles involving denaturation for 10 seconds at 94°C, annealing for 30 seconds at 60°C, and prolongation for 60 seconds at 72°C. All primers used in the PCR procedure were synthesized by GENEWIZ. The primers used to detect STING isoforms are listed in Supplemental Table 1.

### Plasmid construction.

The DNA sequence encoding the mouse or human pmSTING ORF fused with EGFP or 3× Flag, mouse or human erSTING ORF fused with EGFP or 3× Flag, and the sequence encoding the mouse pmSTING ORF with mutation of the TBK1 phosphorylation site (Ser316R) fused with EGFP were synthesized de novo, respectively, and then subcloned into the pcDNA3.1 vector.

### Luciferase and SEAP assays.

HEK93T cells or B16-Blue ISG-KO-STING cells were seeded into 24-well plates at 2 × 10^5^ cells/well for 24 hours. HEK293T cells were transfected with 400 ng h-pmSTING or an h-erSTING plasmid and 800 ng of the IFN promoter luciferase reporter plasmid IFNβ-pGL3 (Addgene) using Lipofectamine 2000 (Invitrogen, Thermo Fisher Scientific) according to the manufacturer’s instructions. B16-Blue ISG-KO-STING cells were transfected with or without si-cGAS or si-SLC19A before transfection with 2 μg pmSTING or erSTING plasmids using Lipofectamine 2000. Six hours after transfection, the medium was replaced with serum-free medium with or without cGAMP (35 μM). After treatment for 8 hours with cGAMP, 20 μL supernatant from B16-Blue ISG-KO-STING cells and 180 μL SEAP substrate (InvivoGen) were added to a 96-well plate. Then, the plate was incubated at 37°C for 5 hours. Absorption at 630 nm was detected on a SpectraMax M5 Multi-Mode Microplate Reader (Molecular Devices). For HEK293T cells, 10 μL supernatant was mixed with luciferin substrate (InvivoGen) in a 1.5 mL Eppendorf tube, and the result was immediately read using a Promega GloMax 20/20. All assays were replicated 3 times.

### Immunofluorescence staining and confocal laser scanning microscopy.

The mouse splenocyte smear samples and human PBMC smear samples were fixed with 2% cool paraformaldehyde (PFA) for 5 minutes and blocked with 1% BSA (Biofrog) for 30 minutes. After washing in cold PBS twice, staining was performed using the primary antibody cocktail containing rabbit anti-STING antibody (ab189430, Abcam,1:200) and fluorochrome-conjugated antibodies against cellular surface markers of different immune cell types (BioLegend) at 4°C in the dark for 30 minutes. After 3 washes in cold PBS plus 0.1% Tween-20 (PBST), the smear samples were stained with Alexa Fluor 594–conjugated goat anti–rabbit IgG (Proteintech, 1:100) at room temperature in the dark for 1 hour. Nuclei were stained with DAPI (Solarbio) at room temperature for 10 minutes. Finally, the smear samples were washed in cold PBST and then sealed with sealing agent (MilliporeSigma).

Flag-, erSTING-Flag–, or pmSTING-Flag–transfected B16-Blue ISG-KO-STING cells or HEK293T cells were fixed in 2% PFA for 5 minutes and blocked with 1% BSA for 30 minutes. The slides were incubated with a primary antibody against Flag (T0053, Affinity, 1:50) at 4°C overnight. Then, the slides were stained with wheat germ agglutinin (WGA) lectin (FITC) (GTX01502, GeneTex, 1:500) for 15 minutes at room temperature in the dark. After washing in cold PBS 3 times, the slides were stained with Alexa Fluor 594–conjugated goat anti–rabbit IgG (Proteintech, 1:100) at room temperature in the dark for 1 hour. Finally, the slides were stained with DAPI and sealed with 50% glycerin as described above. Images were captured using a Nikon C2 confocal microscope.

### Statistics.

Statistical analysis and graph generation were performed with GraphPad Prism 6 (GraphPad Software). All data are presented as the mean ± SD. A 2-tailed Student’s *t* test was used to compare 2 groups of independent samples in the luciferase reporter assays. A 2-way ANOVA was used to determine the variation among or between groups when analyzing the ELISA data. A 1-way ANOVA with Dunnett’s test was used to determine the variation of cGAMP-induced IFN-β production among different antibody-pretreated splenocytes, as detected by ELISA. A *P* value of less than 0.05 was considered statistically significant.

### Study approval.

All animal experiments were performed in accordance with protocols approved by the Harbin Medical University Research Ethics Committee.

## Author contributions

XL and TZ designed experiments and wrote the manuscript with input from all authors. YZ performed experiments and analyzed the data. XZ and XA performed experiments. MW and JS prepared the figures. SW, CL, and SL assisted with data acquisition and analysis. XL and TZ supervised the project.

## Supplementary Material

Supplemental data

## Version 1 12/14/2021

In-Press Preview

## Version 2 02/01/2022

Electronic publication
