# Supplementary material for: An alternatively spliced STING isoform localizes in the cytoplasmic membrane and directly senses extracellular cGAMP
Source: J Clin Invest. 2022 Feb 1;132(3):e144339. doi: 10.1172/JCI144339 (PMC8803335; doi:10.1172/JCI144339)

An alternatively spliced STING isoform localizes in the cytoplasmic membrane and directly senses extracellular cGAMP

Xiaobo Li[1,2,#], Yuanyuan Zhu[1,2,#], Xiao Zhang[1,#], Xiang An[1,#], Mingjiao Weng[1], Jiaqi Shi[2,3], Song Wang[1], Caiqi Liu[2,3], Shengnan Luo[2,3], Tongsen Zheng[2,3,4,*]

Affiliations

1. Department of Pathology, Harbin Medical University, No.157 Baojian Road, Nangang District, Harbin, Heilongjiang, China, 150081;
2. Key Laboratories of Molecular Oncology of Heilongjiang Province, No.150 Haping Road, Nangang District, Harbin, Heilongjiang, China, 150081;
3. Department of Gastrointestinal Medical Oncology, Harbin Medical University Cancer Hospital, No.150 Haping Road, Nangang District, Harbin, Heilongjiang, China, 150081;
4. Department of Phase 1 Trials Center, Harbin Medical University Cancer Hospital, No.150 Haping Road, Nangang District, Harbin, Heilongjiang, China, 150081;

[#]These authors contributed equally to this work.

[*]Corresponding author

Tongsen Zheng, MD, PhD

Department of Gastrointestinal Medical Oncology, Harbin Medical University Cancer Hospital, No.150 Haping Road, Nangang District, Harbin, Heilongjiang, China, 150081,

Phone: 86-0451-86298281,

E-mail: zhengtongsen@hrbmu.edu.cn;

Supplemental Data:

Supplementary table 1. The sequence of primers and siRNAs used in this study.

Primer or siRNA	Sequence (5’->3’)
Mouse Tmem173 forward	GCTGTGCCATGTCCAGTC
Mouse Tmem173 reverse	CAACCGCAAGTACCCAAT
Human TMEM173 forward	TCTCCTCGTCATCATCCAG
Human TMEM173 reserve	AGGAGGATGTTCAGTGCC
negative control	UUCUCCGAACGUGUCACGUTT
	ACGUGACACGUUCGGAGAATT
si-cGAS-mus-660	GGCCGAGACGGUGAAUAAATT
	UUUAUUCACCGUCUCGGCCTT
si-cGAS-mus-892	GGAAAUCCGCUGAGUCAUUTT
	AAUGACUCAGCGGAUUUCCTT
si-cGAS-mus-1032	CCCUGCUGUAACACUUCUUTT
	AAGAAGUGUUACAGCAGGGTT
si-SLC19A1-1	CCAAGGAGCAGGUGACUAATT
	UUAGUCACCUGCUCCUUGGTT
si-SLC19A1-2	UAAGUGAACUGGUGGAAAATT
	UUUUCCACCAGUUCACUUATT
si-SLC19A1-3	CCAGGAAACUAGAUCGCAUTT
	AUGCGAUCUAGUUUCCUGGTT

Supplemental Figures

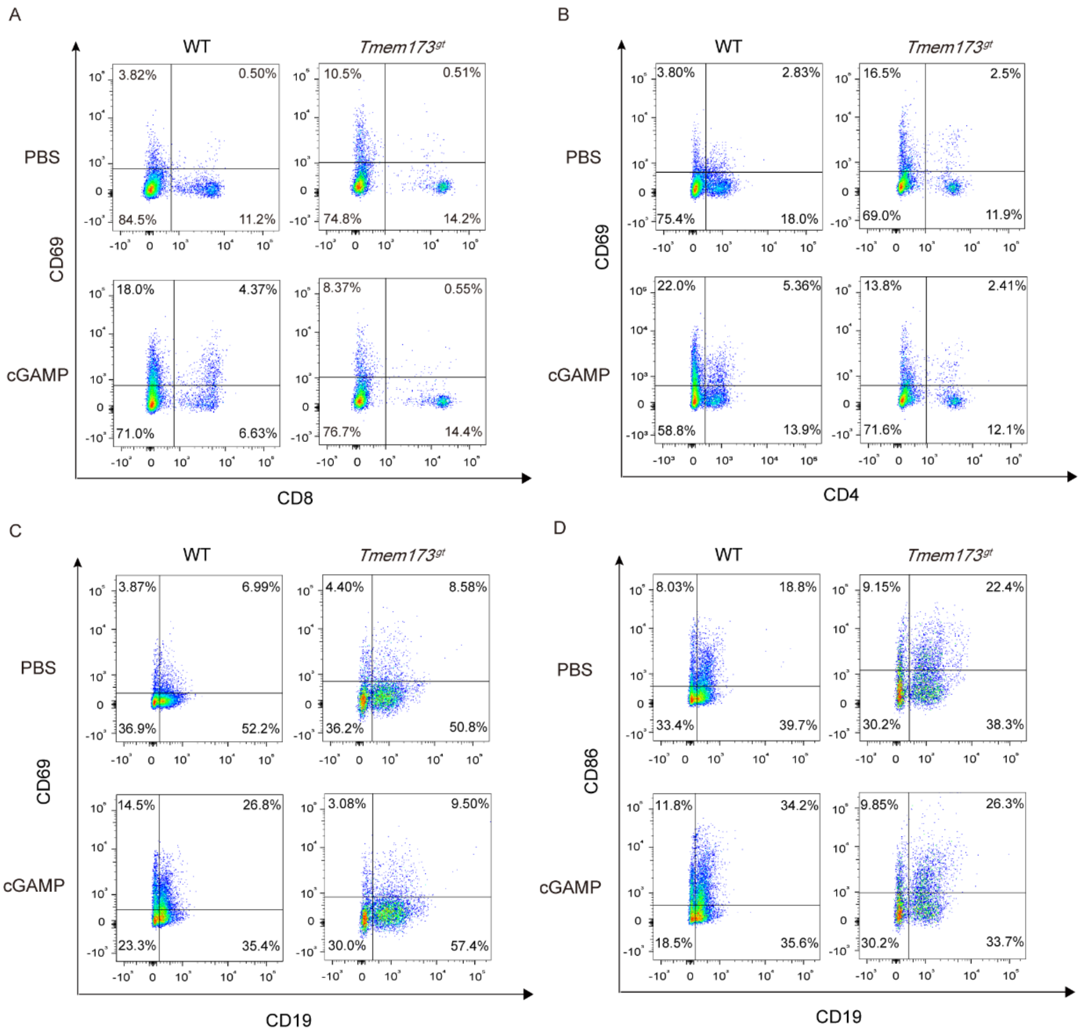

Supplemental Figure 1. Extracellular cGAMP activates T and B lymphocytes. Related to Figure 1. (A) The expression of CD69 was detected by flowcytometry in *WT* and *Tmem173^{gt}* CD4+ T cells treated with vehicle or cGAMP. (B) The expression of CD69 was detected by flowcytometry in *WT* and *Tmem173^{gt}* CD8+ T cells treated with vehicle or cGAMP. (C) The expression of CD69 was detected by flowcytometry in *WT* and *Tmem173^{gt}* B cells treated with vehicle or cGAMP. (D) The expression of CD86 was detected by flowcytometry in *WT* and *Tmem173^{gt}* B cells treated with vehicle or cGAMP.

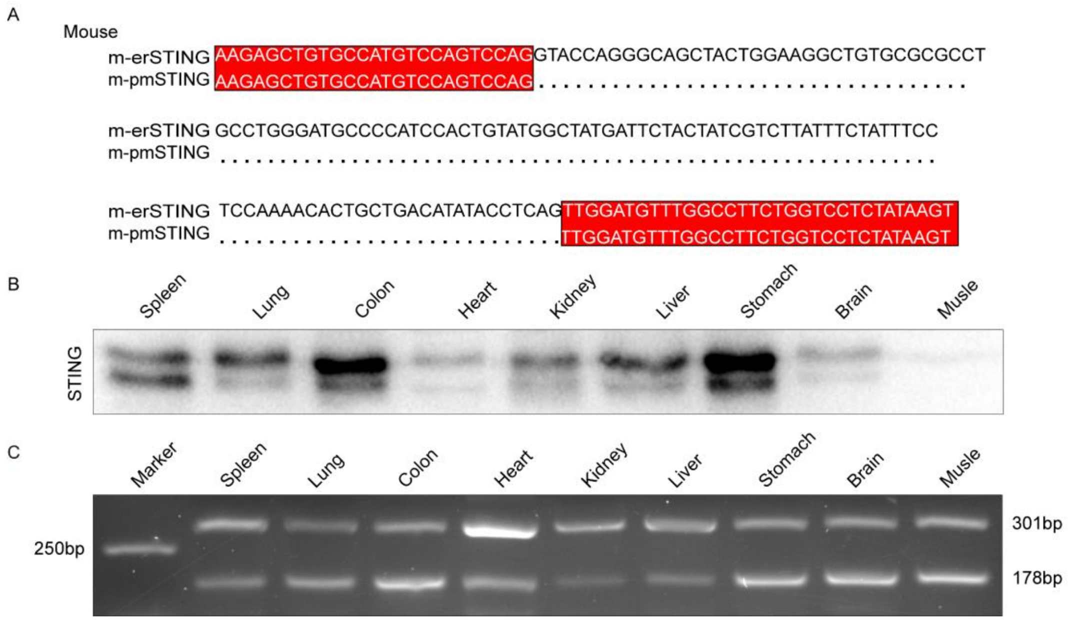

Supplemental Figure 2. An alternatively spliced isoform of mouse STING. Related to Figure 3. (A) cDNA sequence of both STING isoforms in mice splenocytes. (B) Both STING isoforms have been detected by Western Blot in different tissues of mice. (C) Both STING isoforms have been validated by PCR in different tissues of mice.

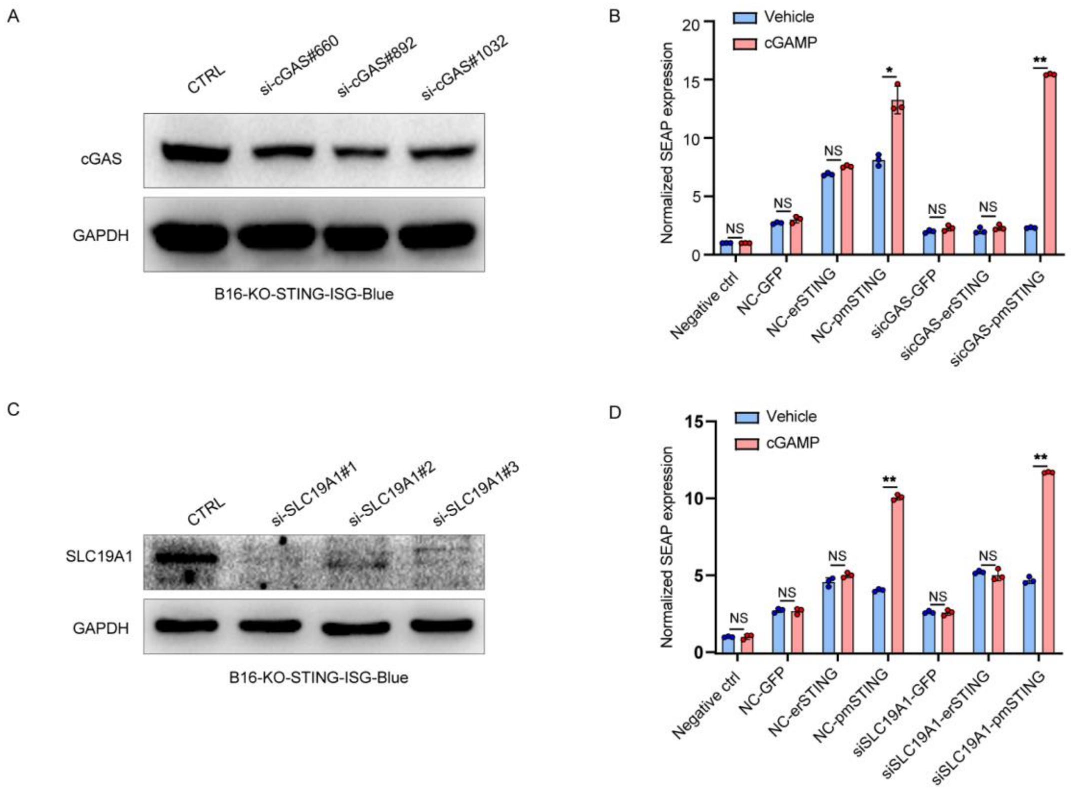

Supplemental Figure 3. Mouse pmSTING directly senses extracellular cGAMP and promotes IFN production. Related to Figure 4. (A) The expression of cGAS in $B16^{Tmem173-/-}$ cells transfected with three different siRNAs targeting cGAS was detected by western blot. (B) $B16^{Tmem173-/-}$ cells stably expressing alkaline phosphatase to monitor interferon activity were transfected with erSTING-FLAG or pmSTING-FLAG after knocking down cGAS. The alkaline phosphatase was detected in these cells after treated with vehicle or cGAMP (n=3). ** represents $p<0.01$; NS means not significant, by 2-tailed, paired Student's *t* test. (C) The expression of SLC19A1 in $B16^{Tmem173-/-}$ cells transfected with three different siRNAs targeting SLC19A1 was detected by western blot. (D) $B16^{Tmem173-/-}$ cells stably expressing alkaline phosphatase to monitor interferon activity were transfected with erSTING-FLAG or pmSTING-FLAG after knocking down SLC19A1. The alkaline phosphatase was detected in these cells after treated with vehicle or cGAMP (n=3). * represents $p<0.05$, ** represents $p<0.01$, NS means not significant, by 2-tailed, paired Student's *t* test.

Human

```
h-erSTING  CGTAGCGCCGGGCCTTCTCTCCTCGTCATCATCCAGAGCAGCCAGTGTCCGGGAGGCAGA
h-pmSTING  CGTAGCGCCGGGCCTTCTCTCCTCGTCATCATCCAGAGCAGCCAGTGTCCGGGAGGCAGA

h-erSTING  AGATGCCCACTCCAGCCTGCATCCATCCATCCCGTGTCCCAGGGGTCACGGGGCCCAGA
h-pmSTING  AG..........................................................

h-erSTING  AGGCAGCCTTGGTTCTGCTGAGTGCCTGCCTGGTGACCCTTTGGGGGCTAGGAGAGCCAC
h-pmSTING  ............................................................

h-erSTING  CAGAGCACACTCTCCGGTACCTGGTGCTCCACCTAGCCTCCCTGCAGCTGGGACTGCTGT
h-pmSTING  ............................................................

h-erSTING  TAAACGGGGTCTGCAGCCTGGCTGAGGAGCTGCGCCACATCCACTCCAGGTACCGGGGCA
h-pmSTING  .................................................GTACCGGGGCA

h-erSTING  GCTACTGGAGGACTGTGCGGGCCTGCCTGGGCTGCCCCCTCCGCCGTGGGGCCCTGTTGC
h-pmSTING  GCTACTGGAGGACTGTGCGGGCCTGCCTGGGCTGCCCCCTCCGCCGTGGGGCCCTGTTGC
```

Supplemental Figure 4. The cDNA sequence of both human STING isoforms in PBMCs. Related to figure 6.

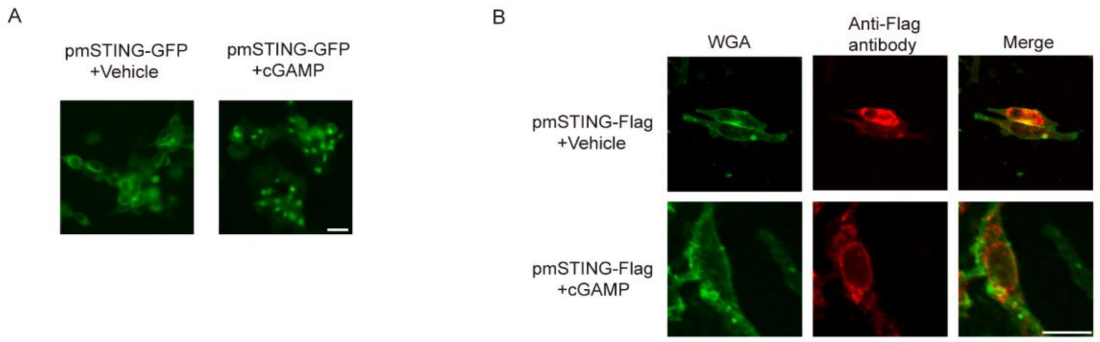

Supplemental Figure 5. Mouse pmSTING translocates from plasma membrane into the cytoplasm upon stimulating by the extracellular cGAMP. (A) The distribution of pmSTING-EGFP in 293T cells with or without extracellular cGAMP treatment using confocal microscopy. Scale bars: 50 µm. (B) B16$^{Tmem173\text{-/-}}$cells were transfected with pmSTING-FLAG and treated with or without cGAMP. An antibody against FLAG was used to detect the distribution of pmSTING-FLAG in B16$^{Tmem173\text{-/-}}$ cells using confocal microscopy. Scale bars: 20 µm.

Full unedited gel for Figure 1

Figure 1E

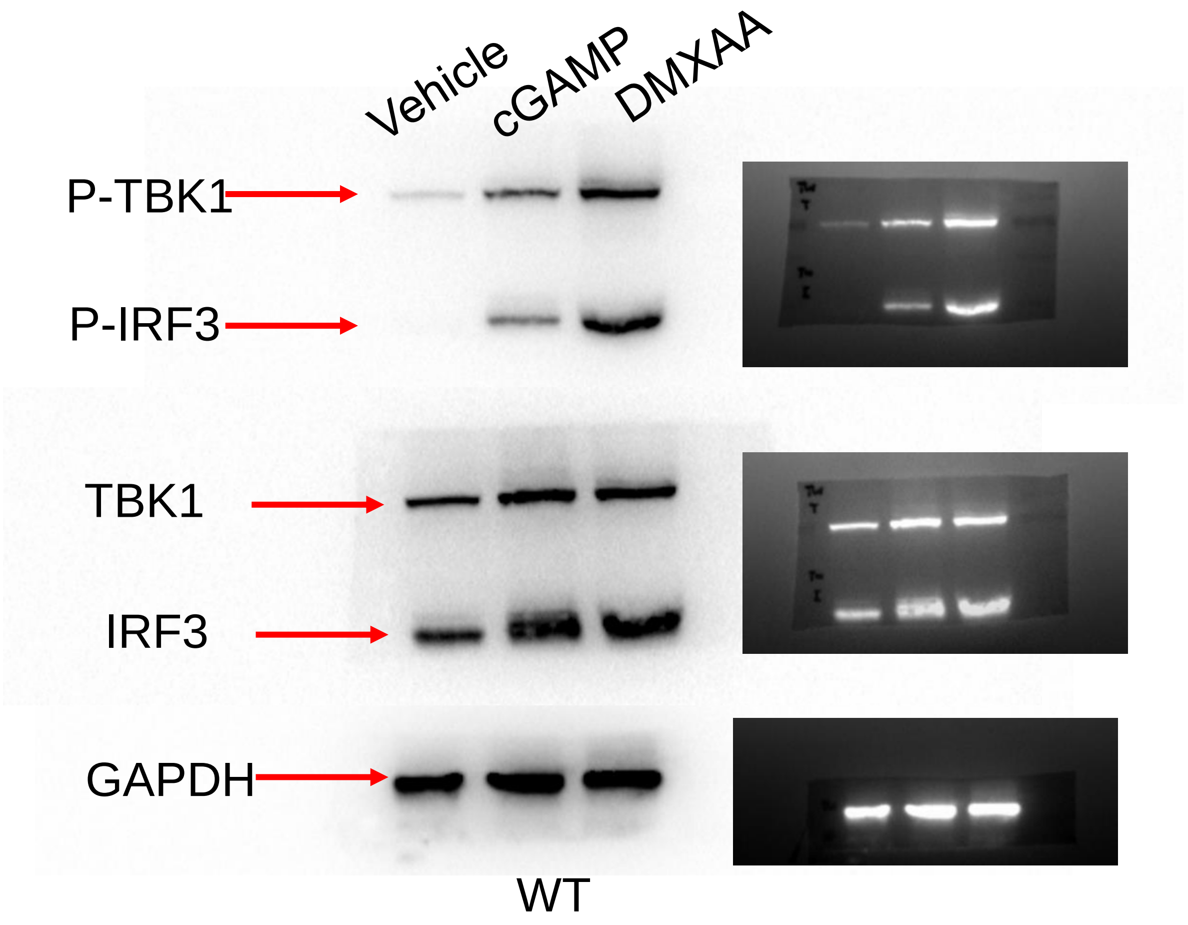

Full unedited gel for Figure 1

Figure 1E

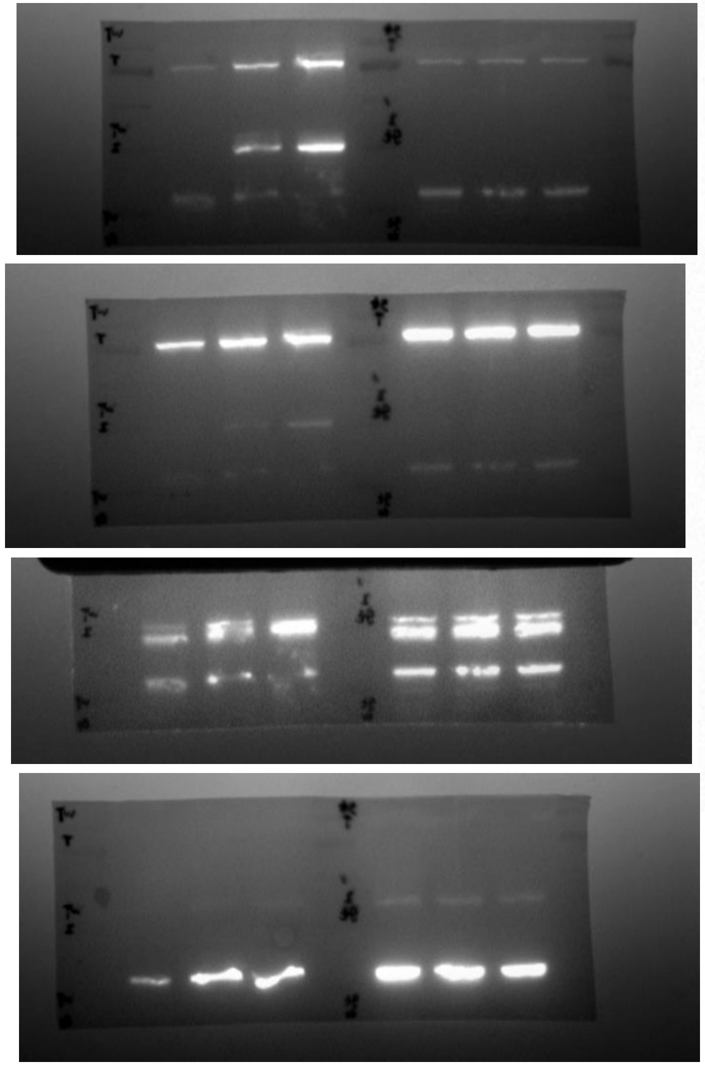

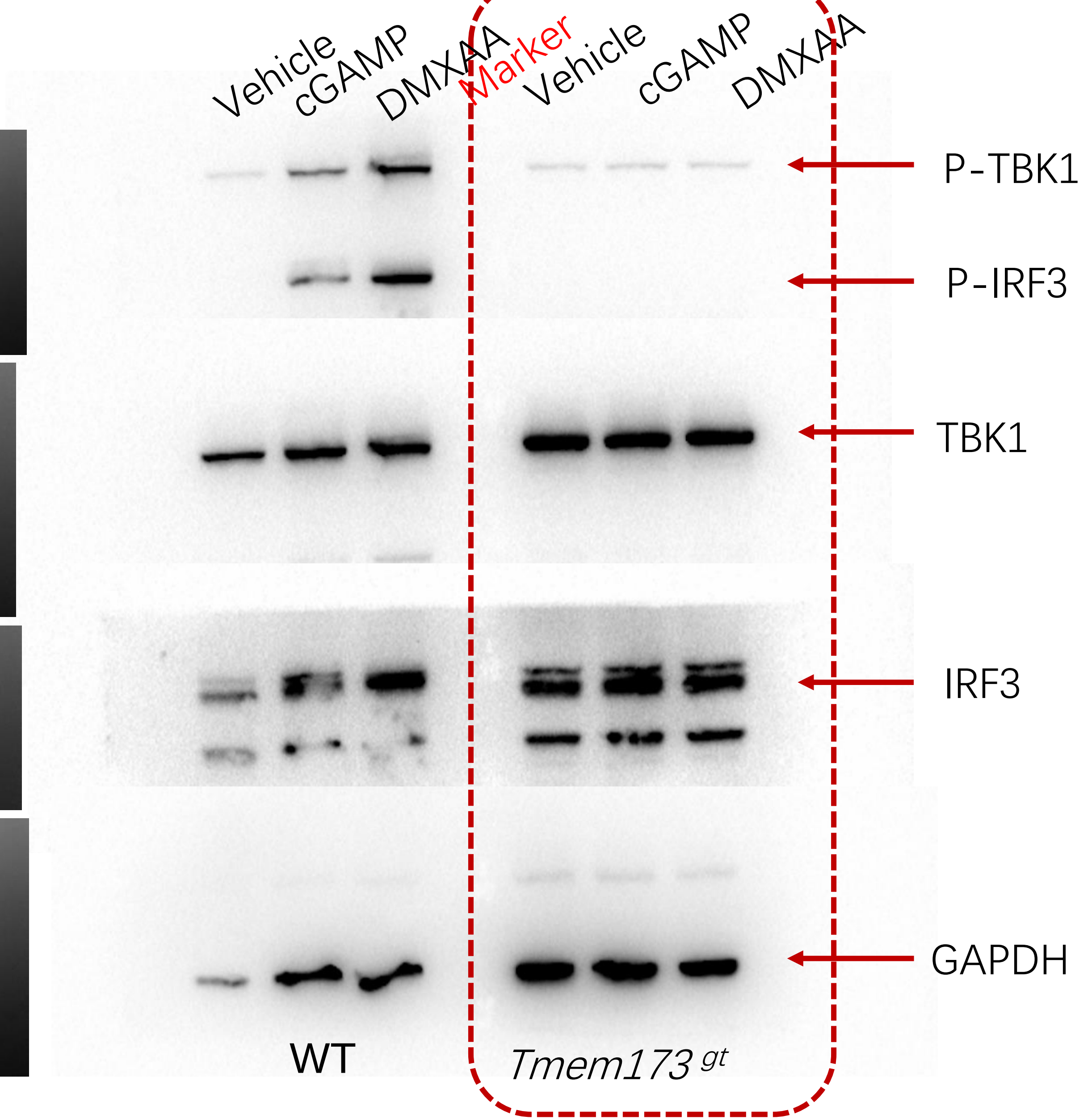

Full unedited gel for Figure 2

Figure 2B

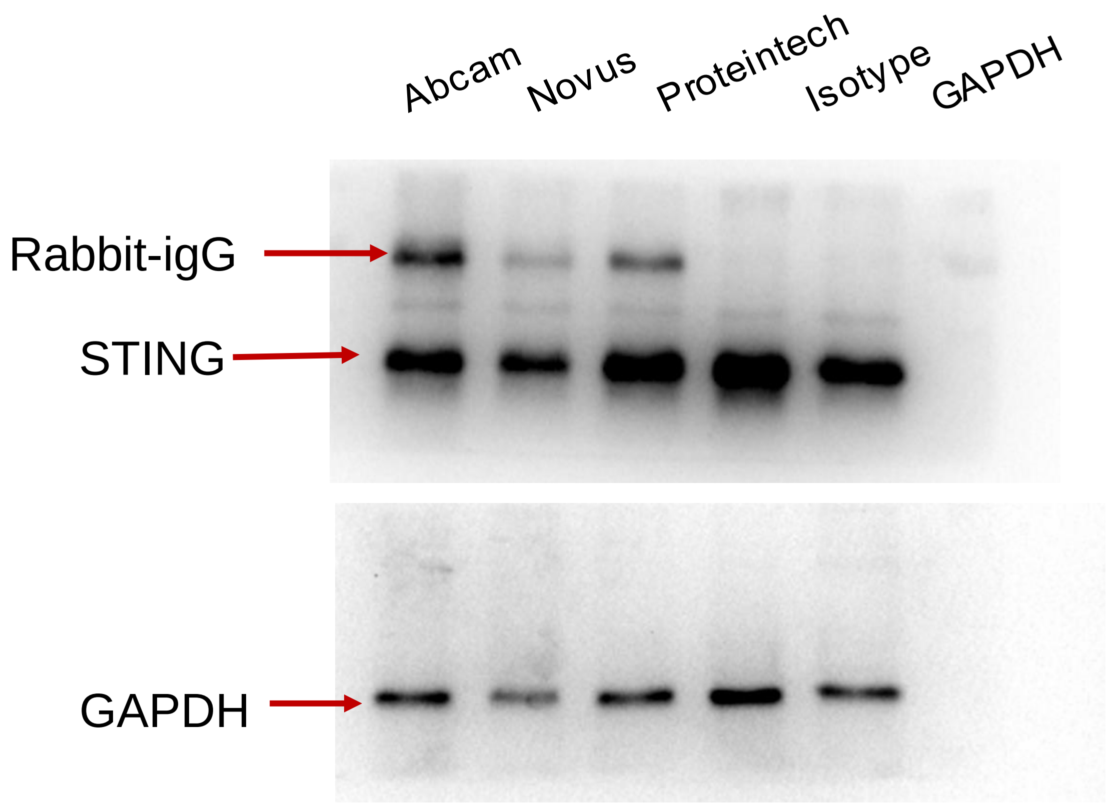

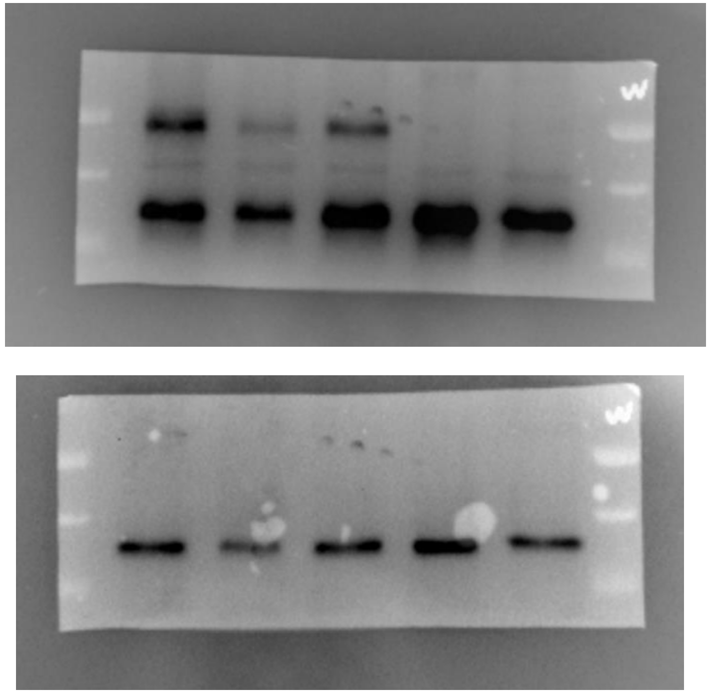

Mouse Spleenocyte

Full unedited gel for Figure 3

Figure 3B

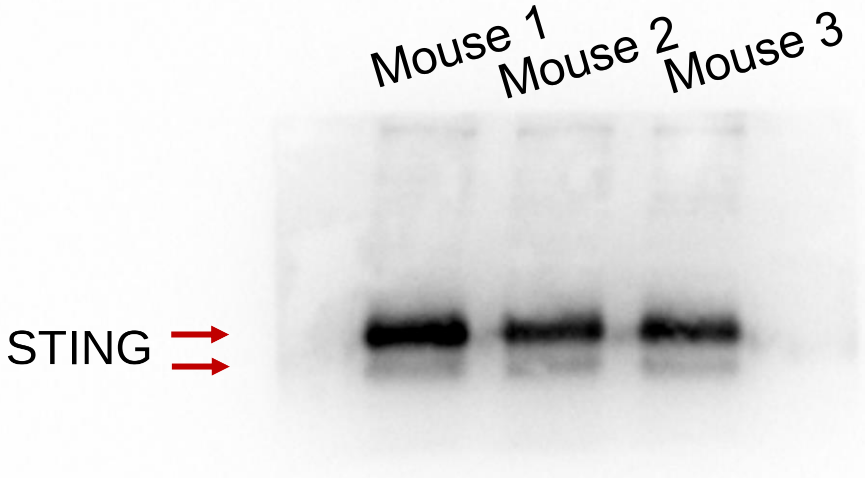

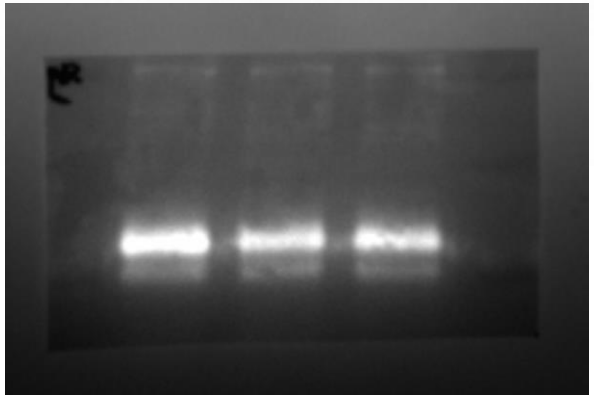

Figure 3C

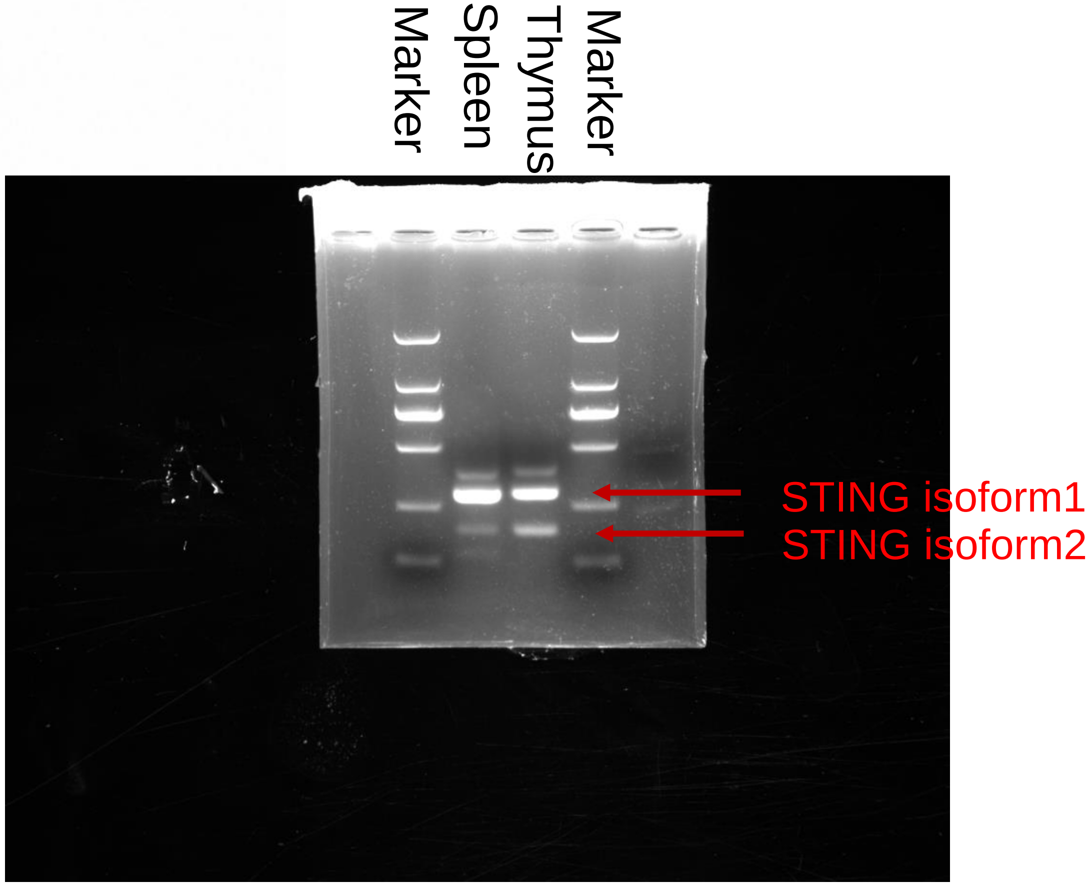

Full unedited gel for Figure 3

Figure 3E

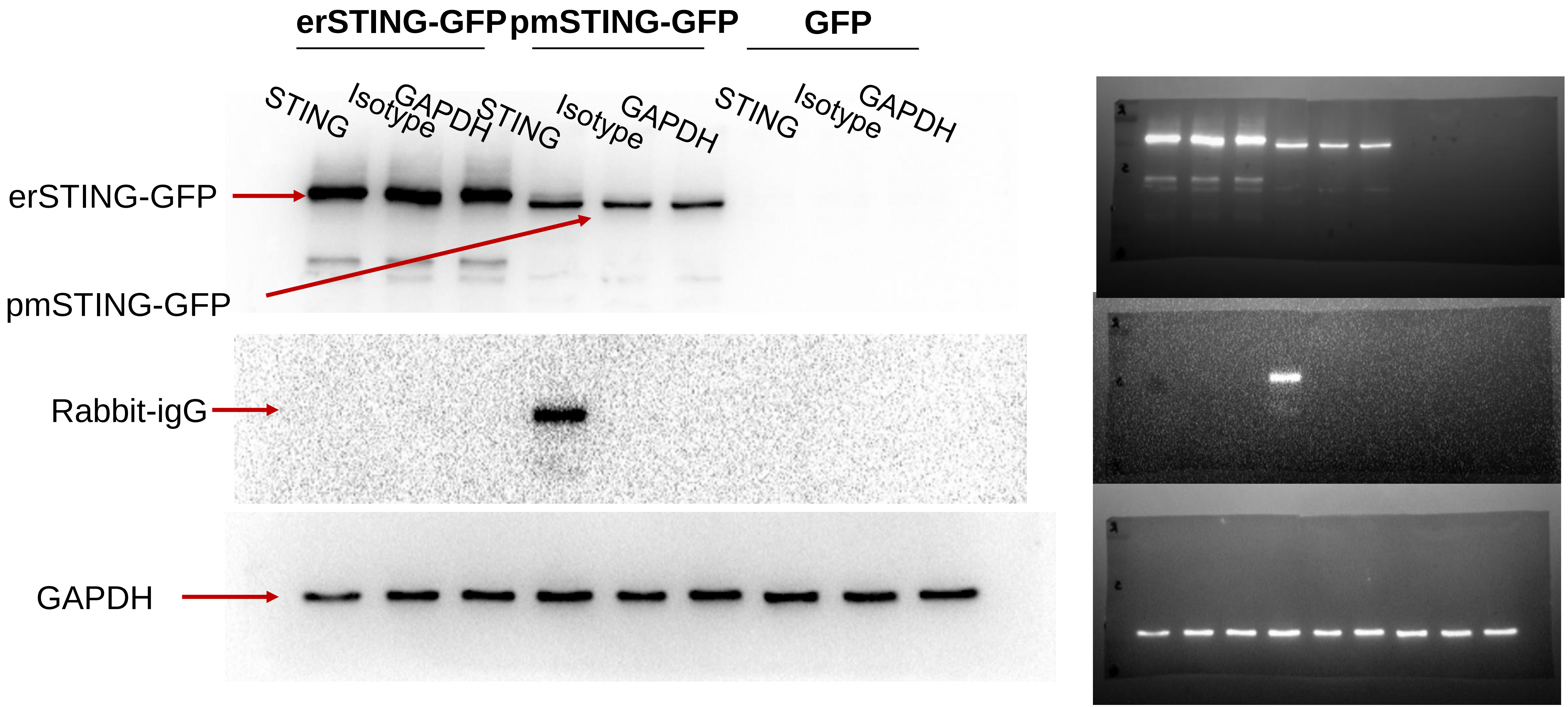

Full unedited gel for Figure 4

Figure 4B

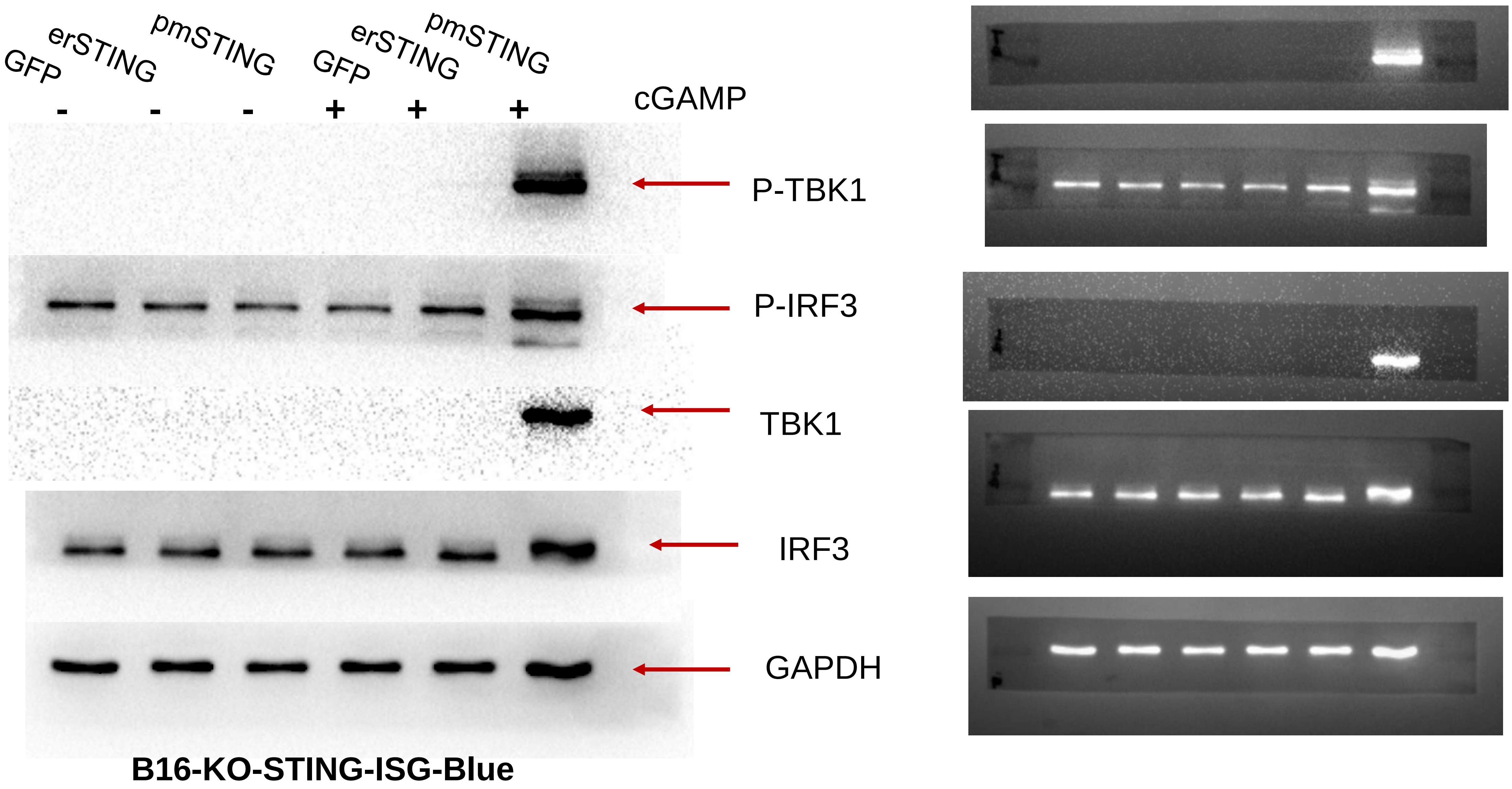

Full unedited gel for Figure 4

Figure 4C

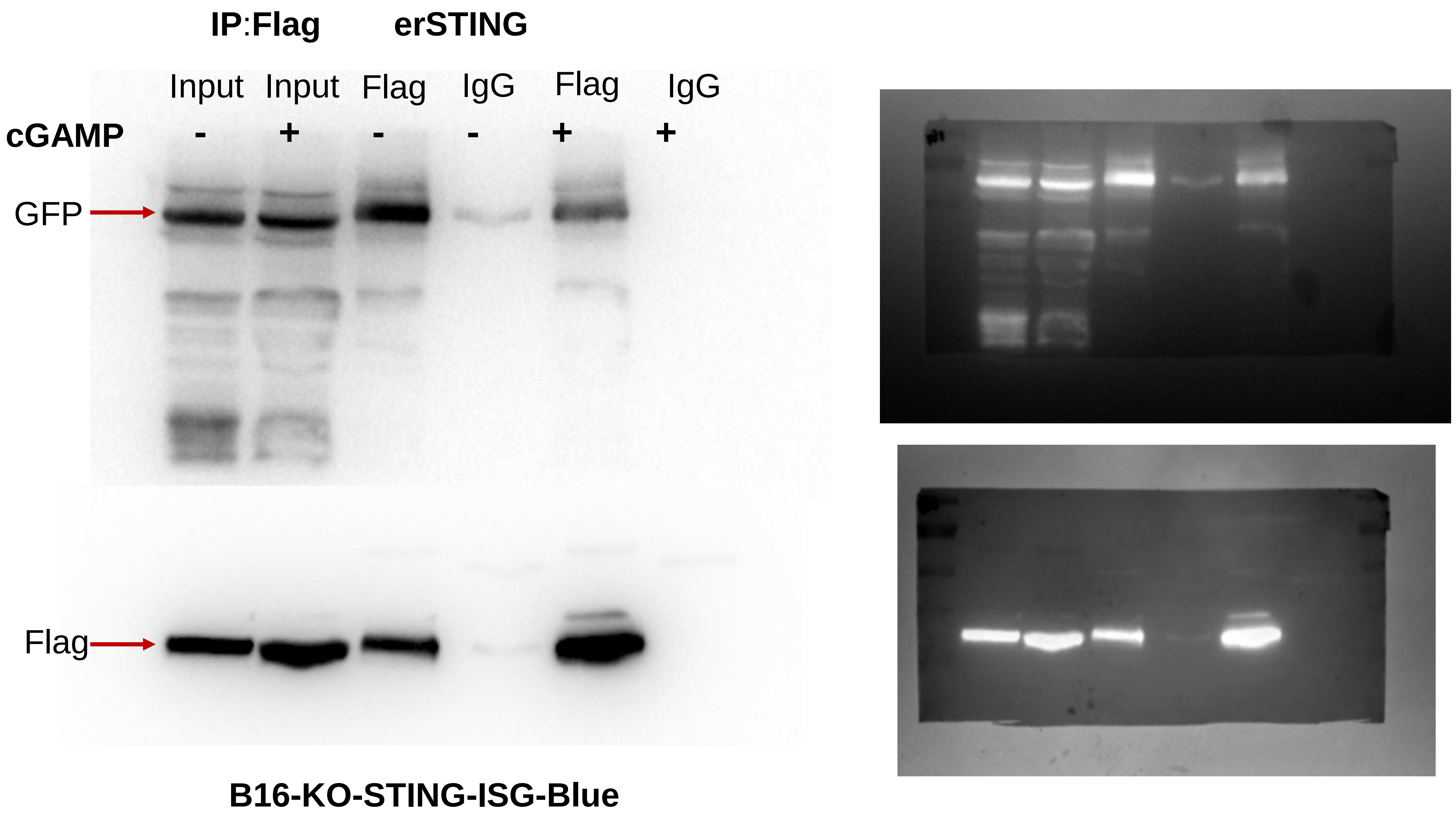

Full unedited gel for Figure 4

Figure 4C

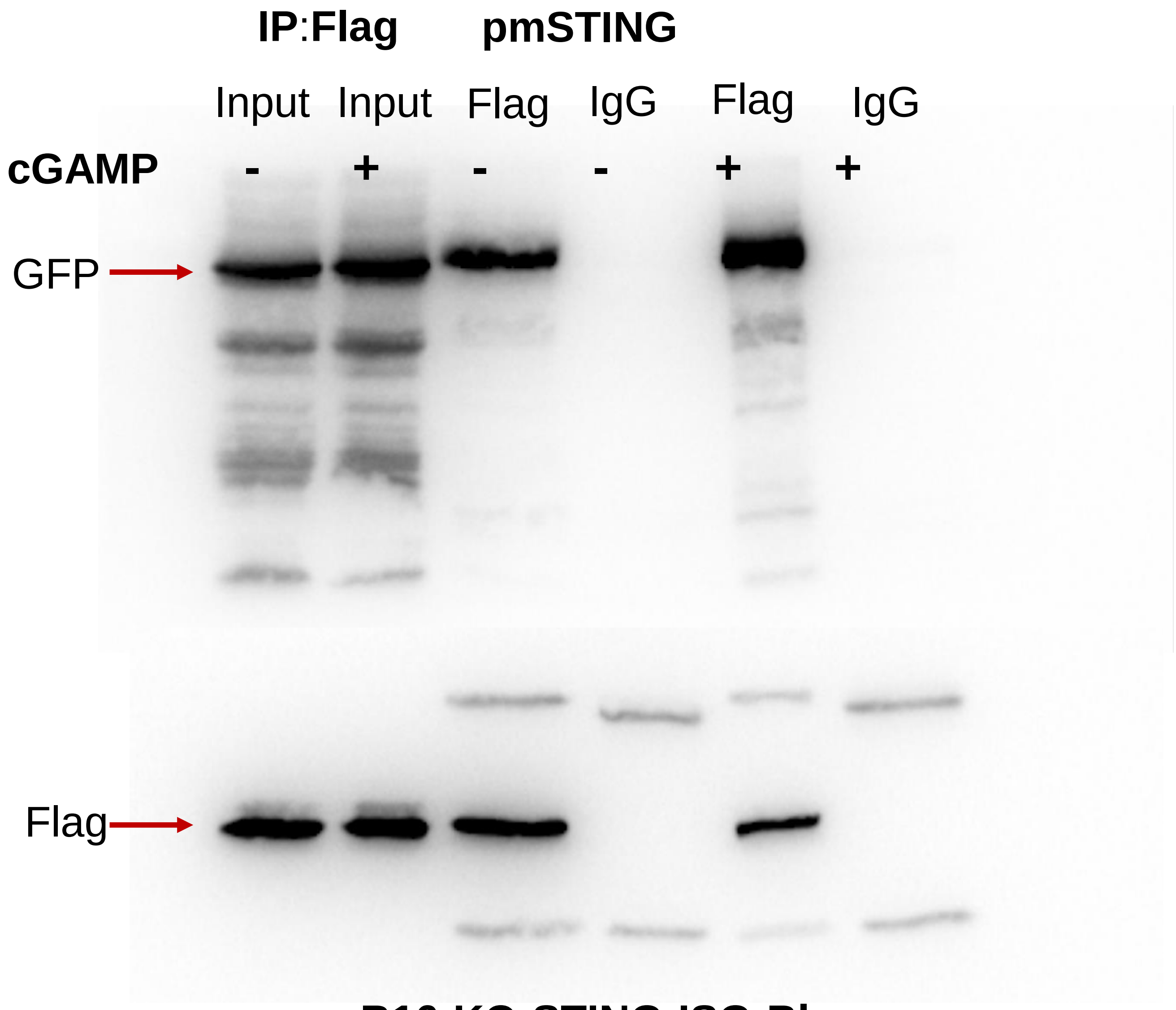

B16-KO-STING-ISG-Blue

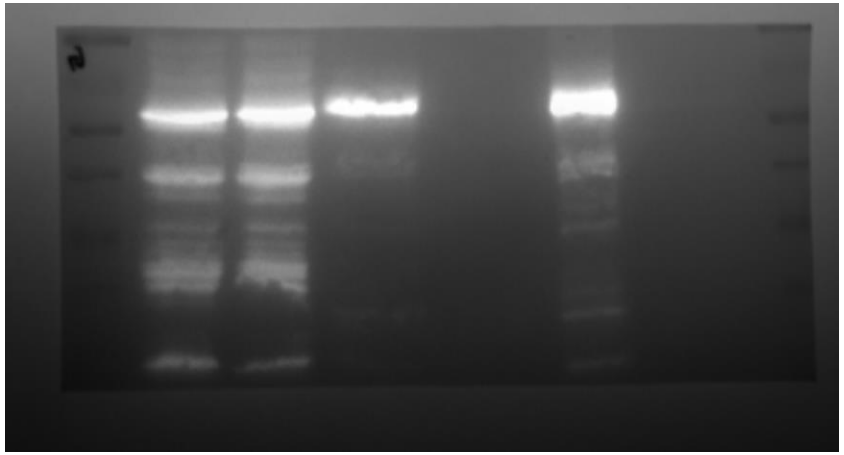

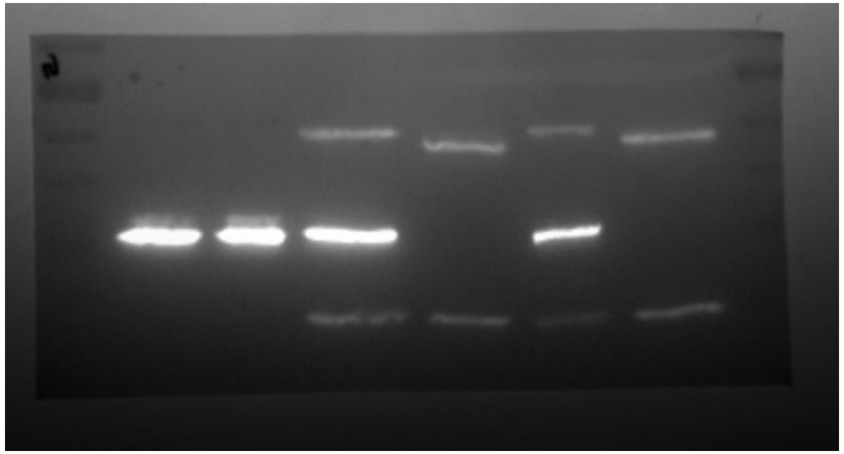

Full unedited gel for Figure 4

Figure 4D

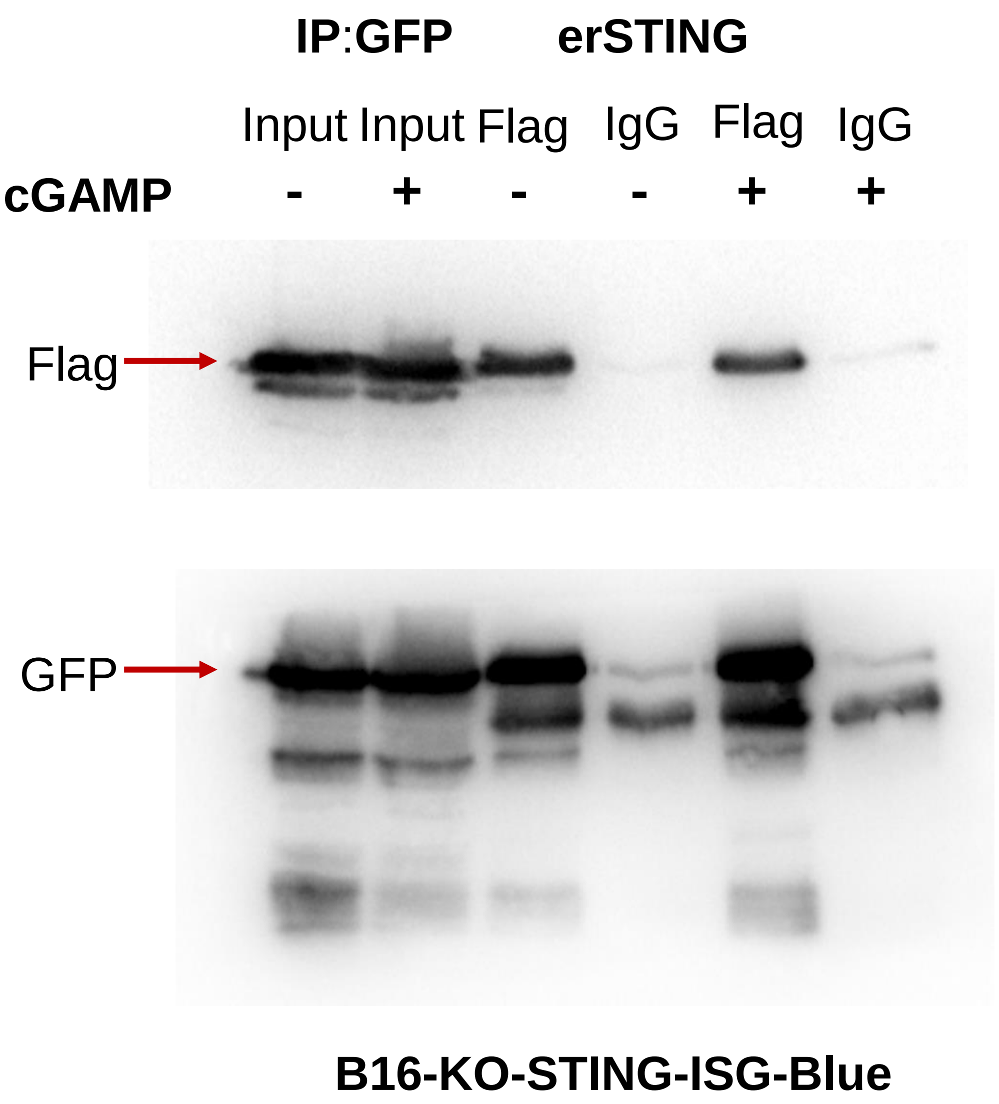

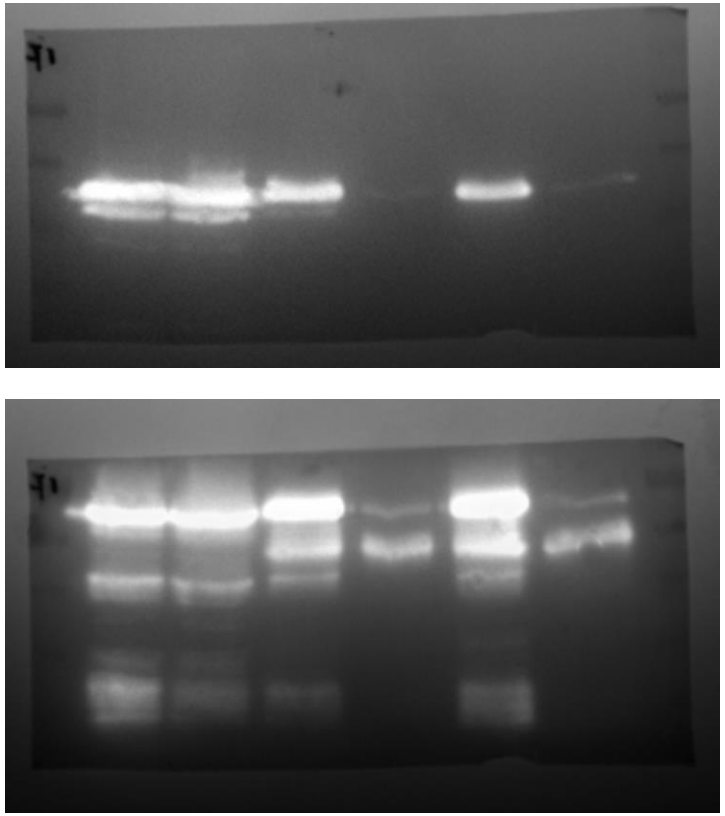

Full unedited gel for Figure 4

Figure 4D

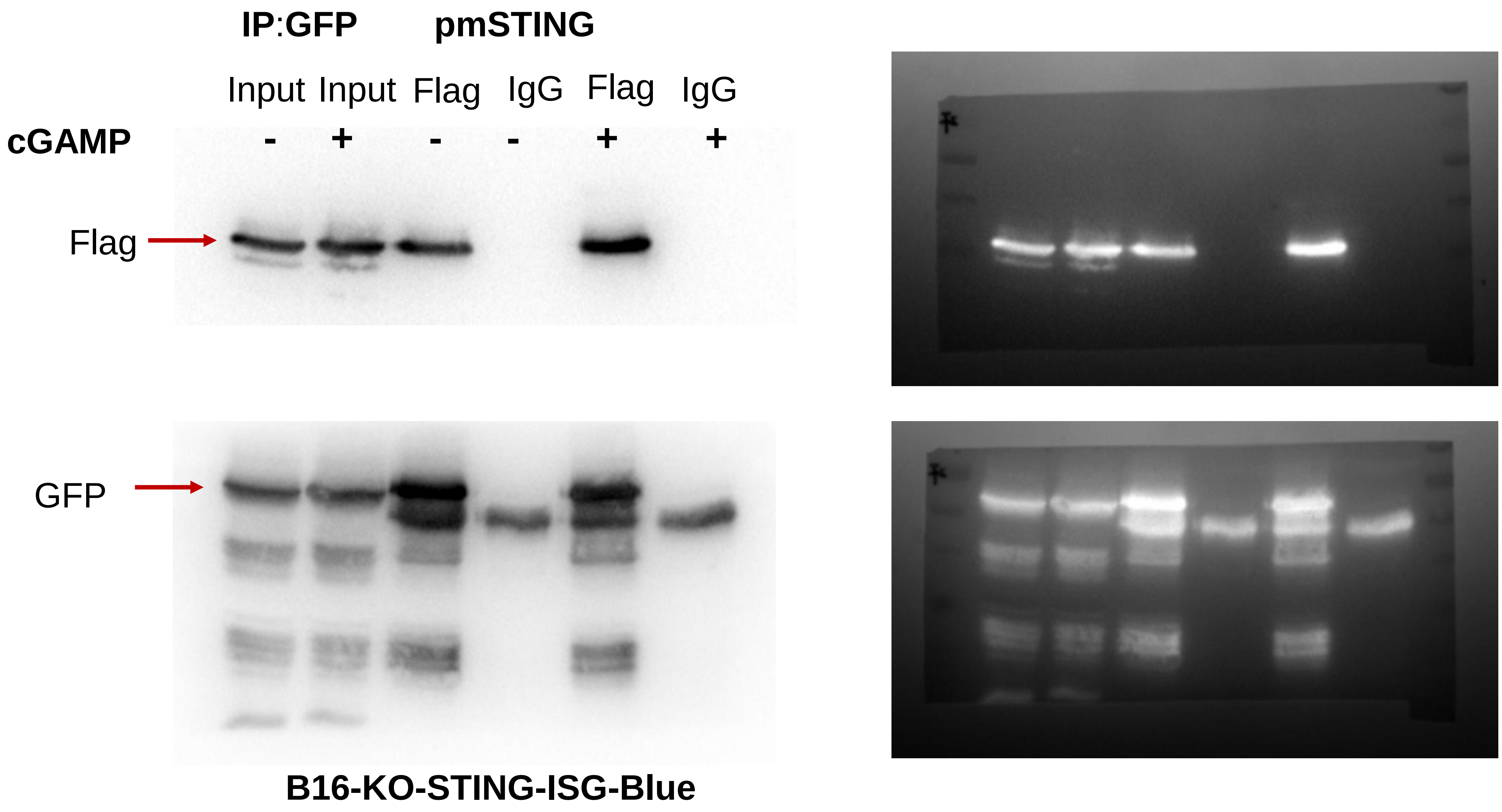

Full unedited gel for Figure 4

Figure 4E

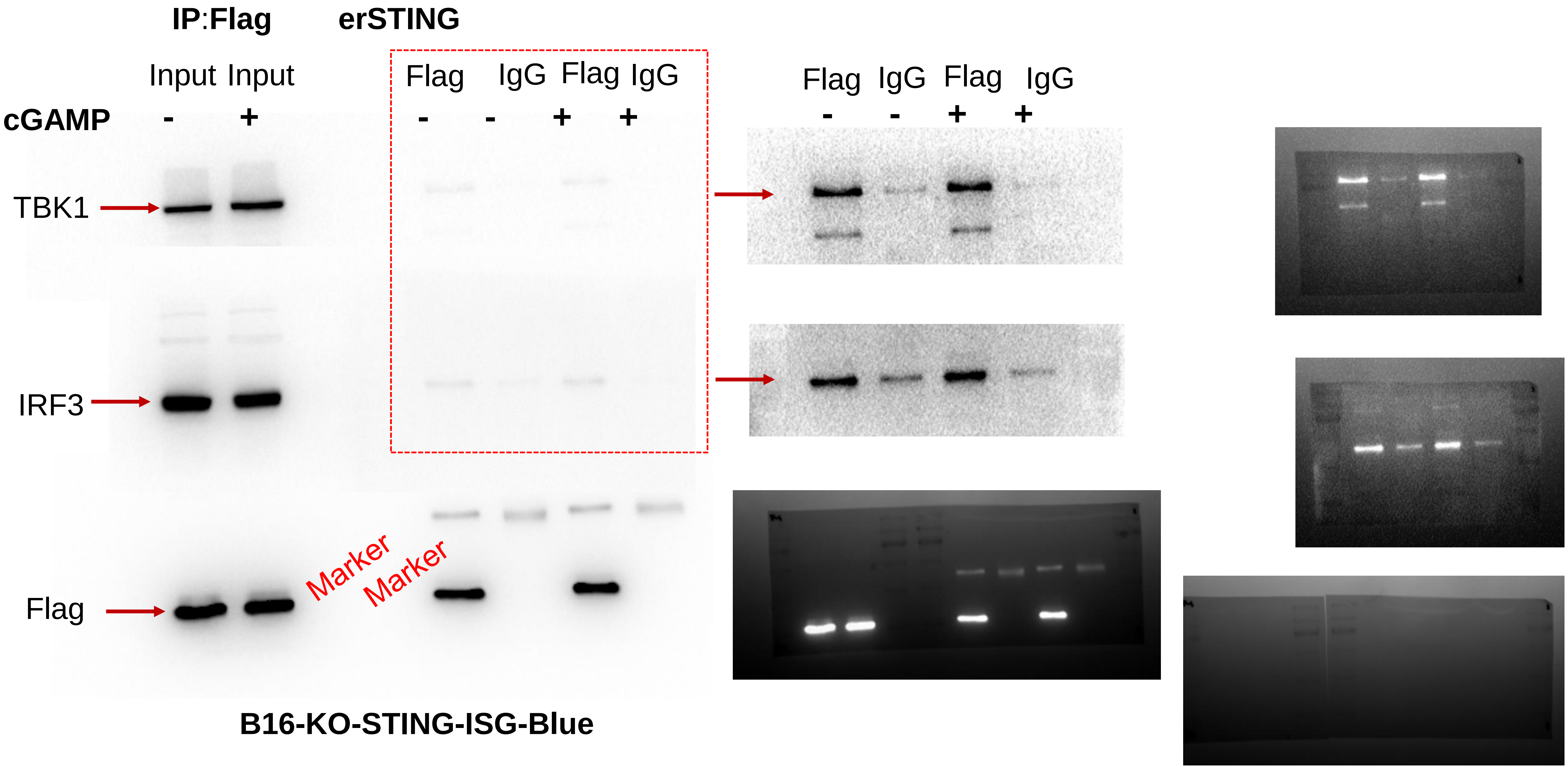

Full unedited gel for Figure 4

Figure 4E

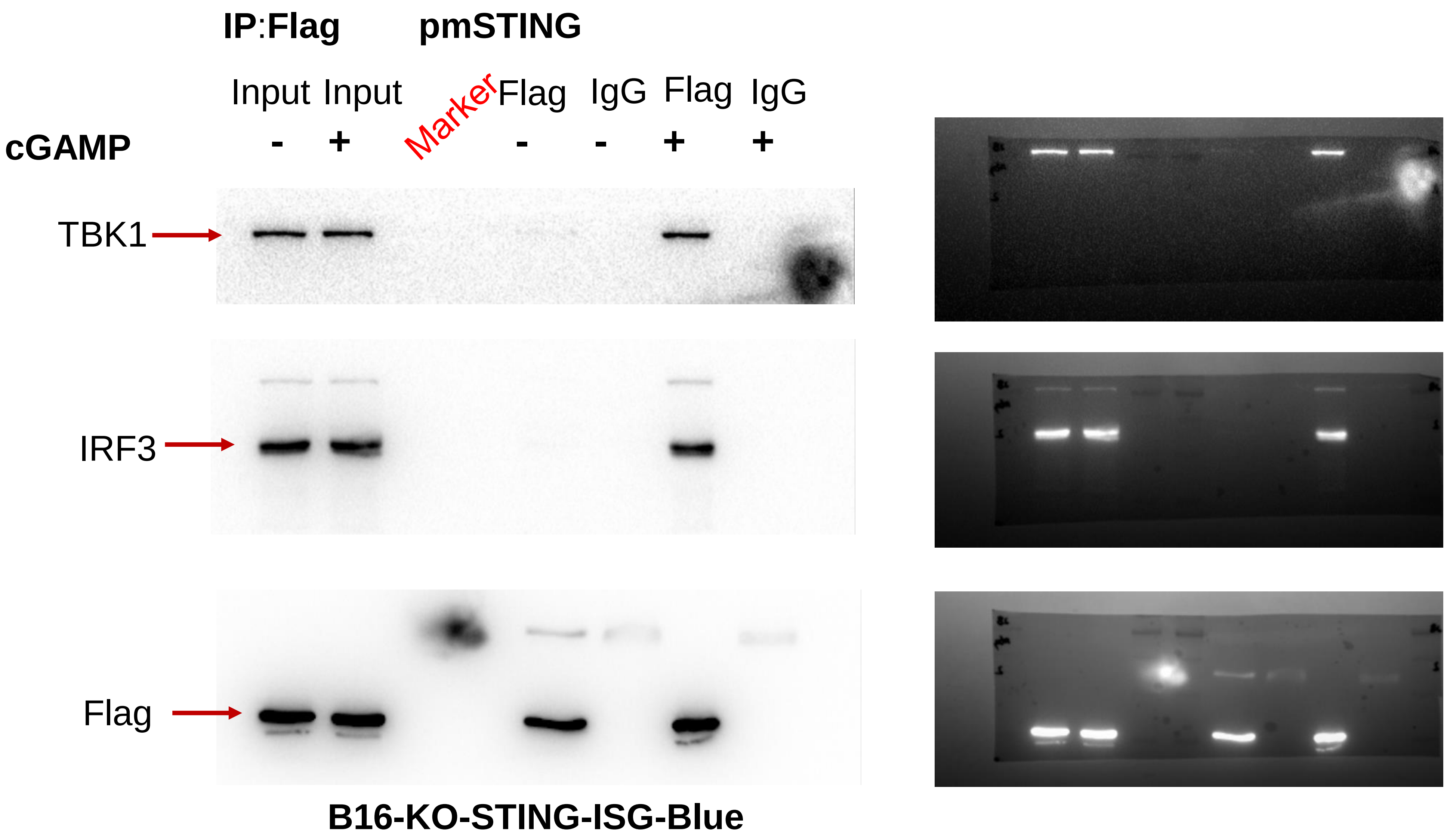

Full unedited gel for Figure 5

Figure 5B

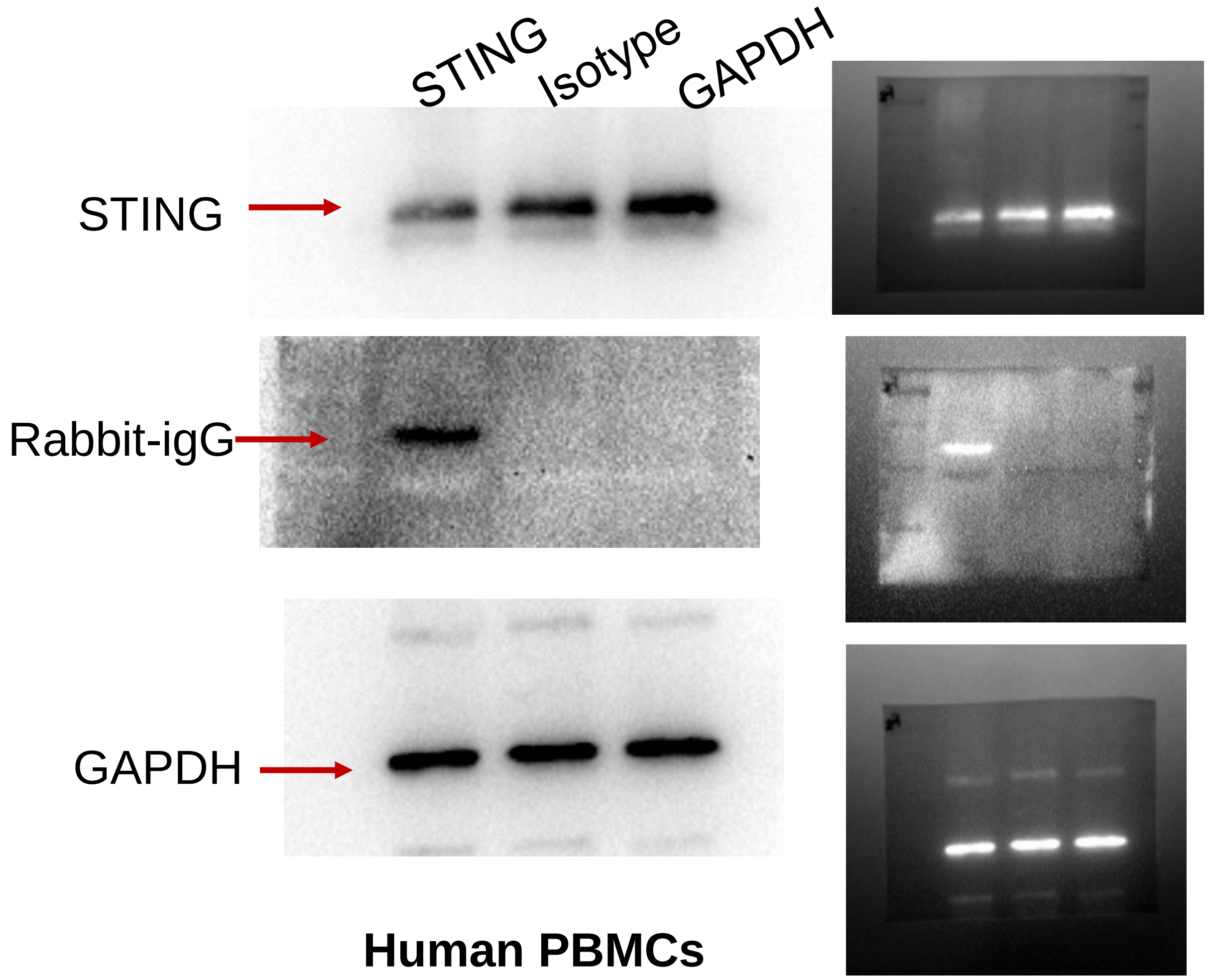

Figure 5F

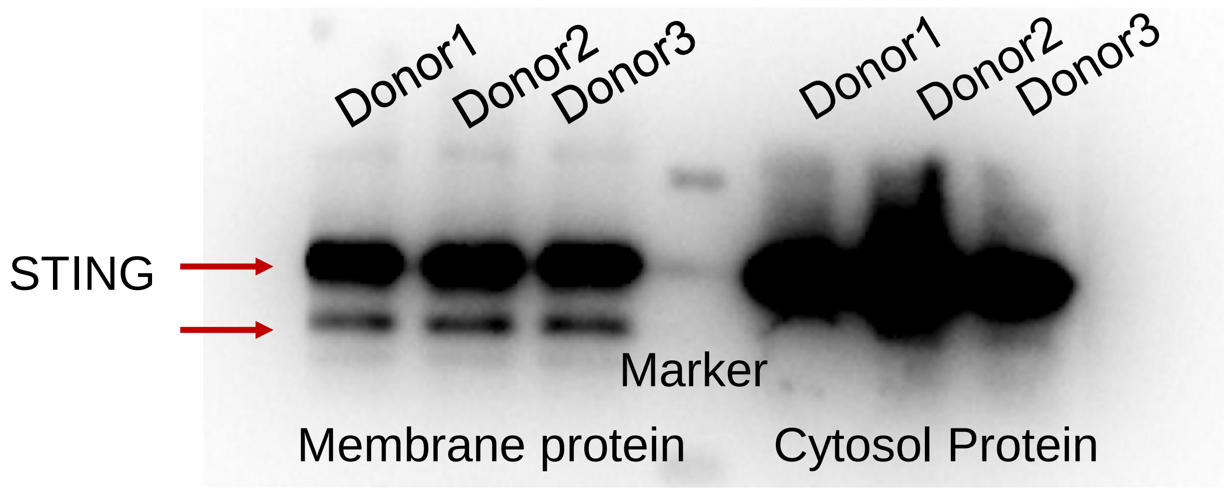

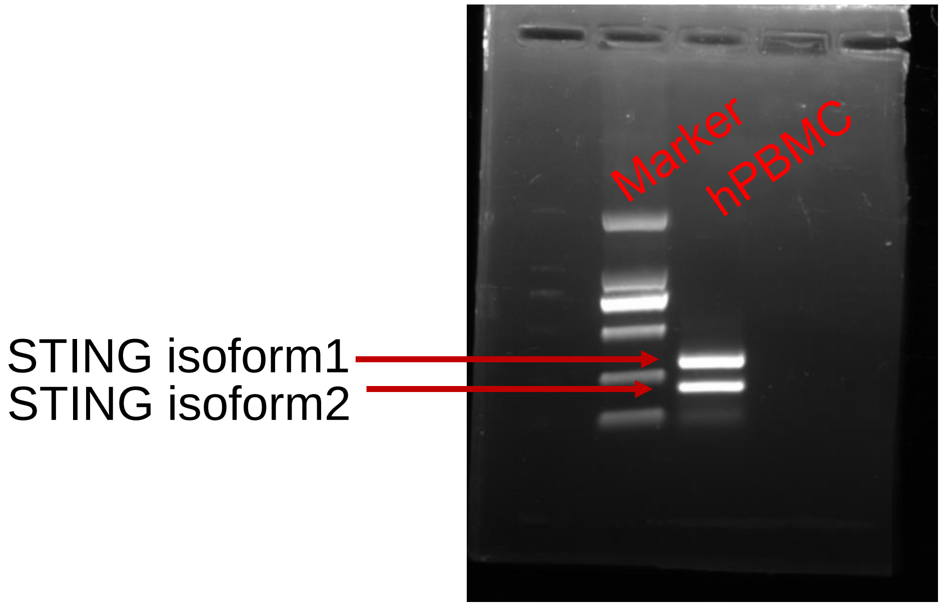

Figure 5G

Full unedited gel for Figure 6

Figure 6B

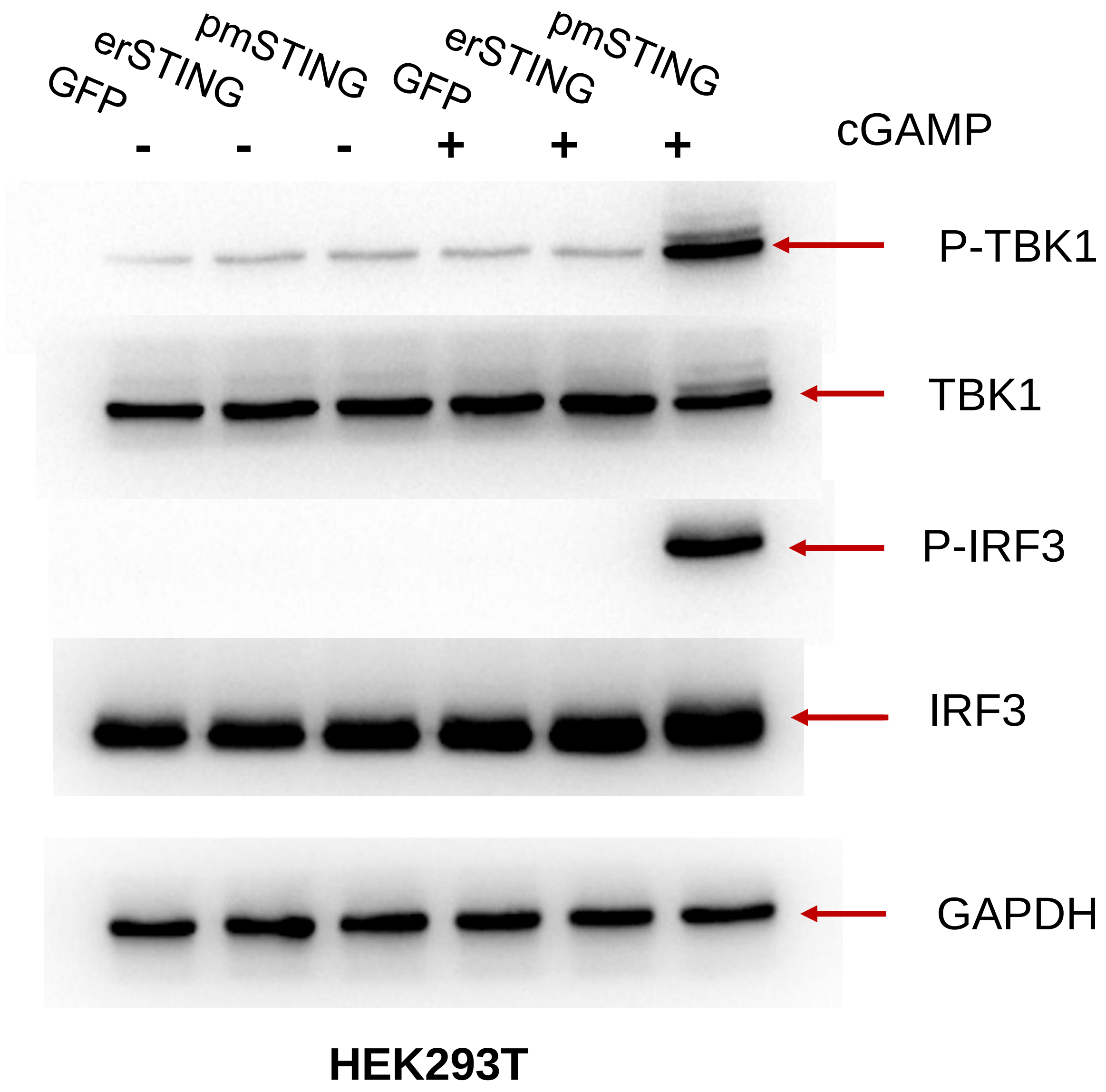

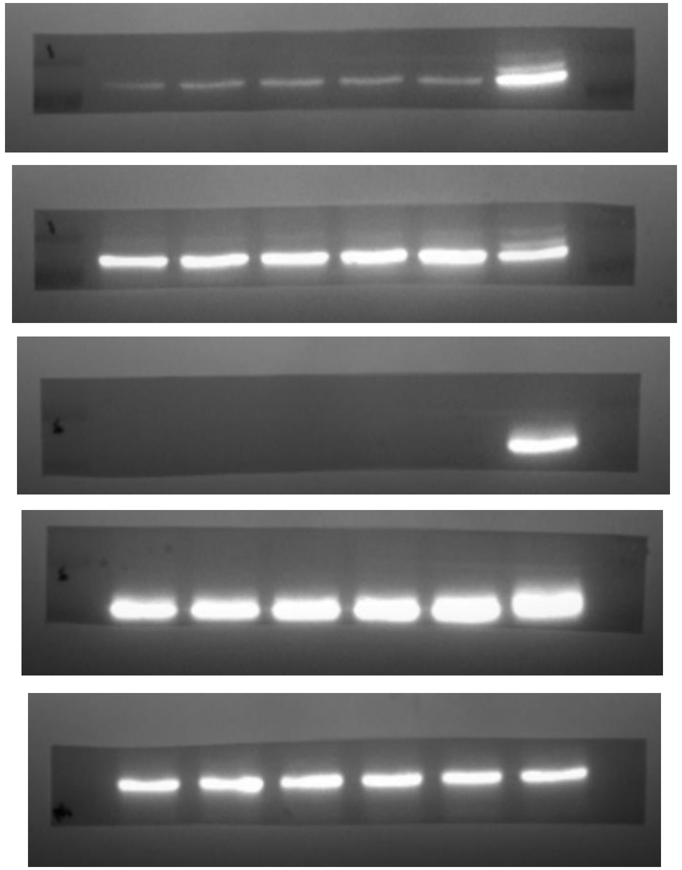

Full unedited gel for Figure 6

Figure 6C

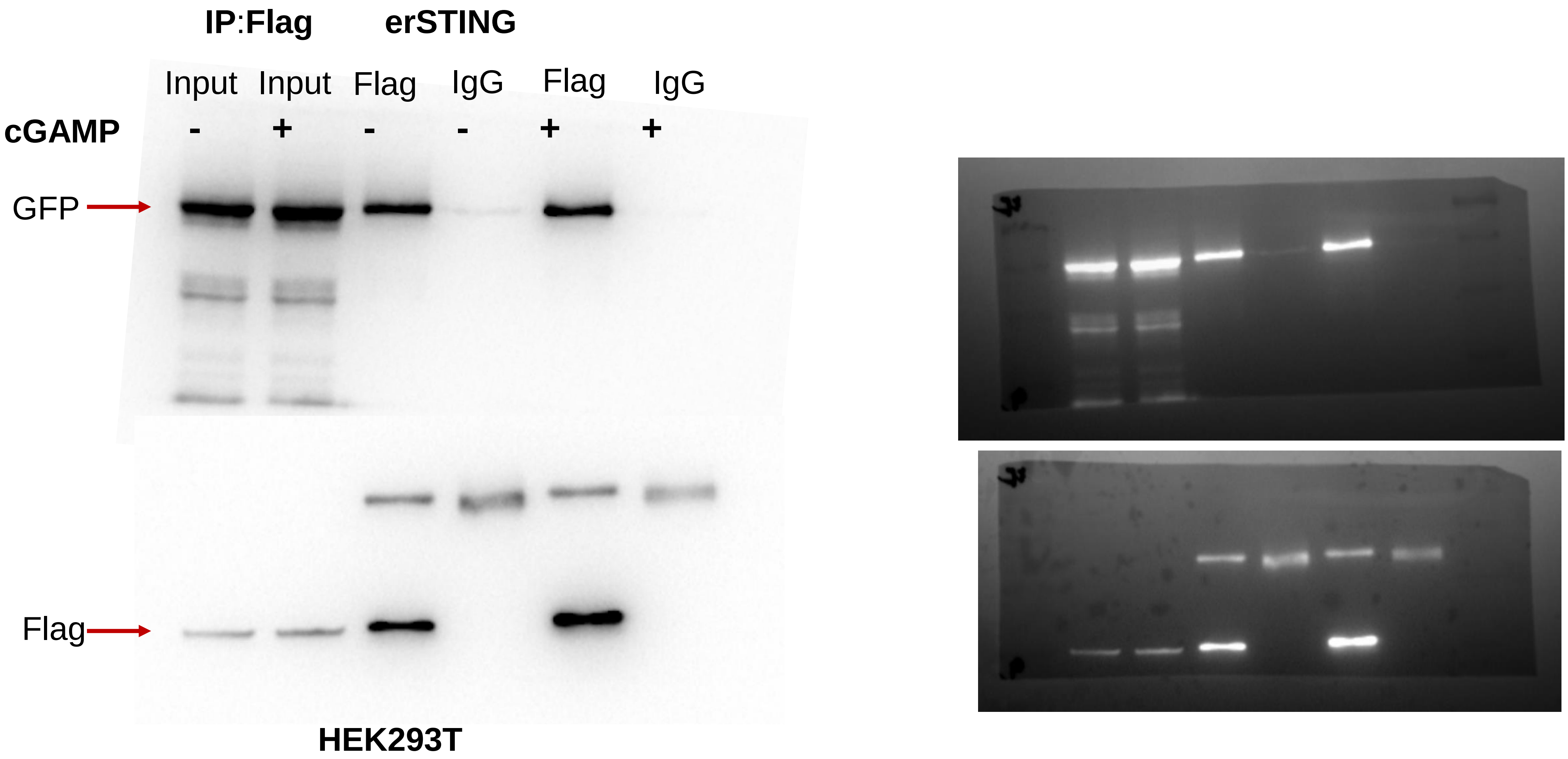

Full unedited gel for Figure 6

Figure 6C

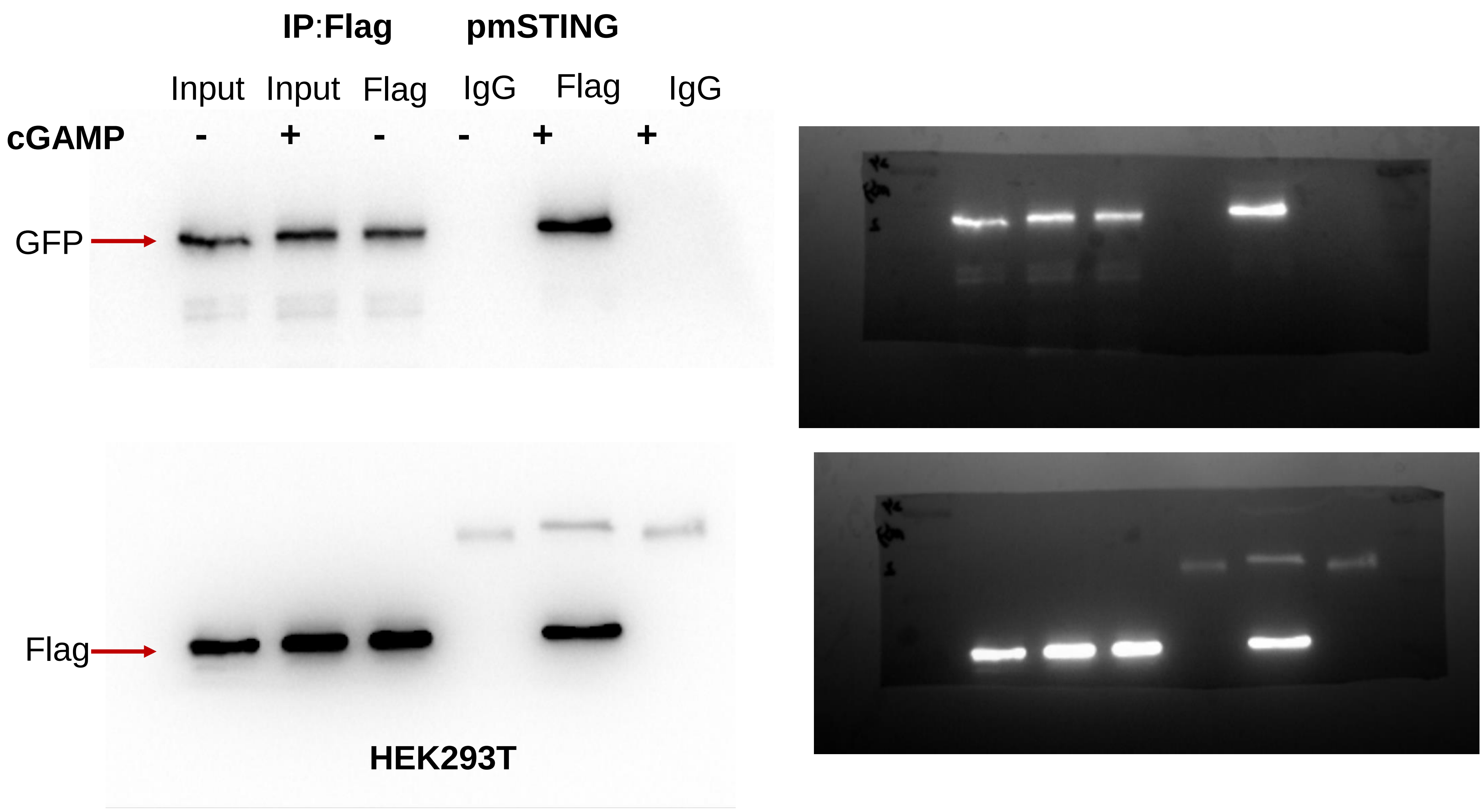

Full unedited gel for Figure 6

Figure 6D

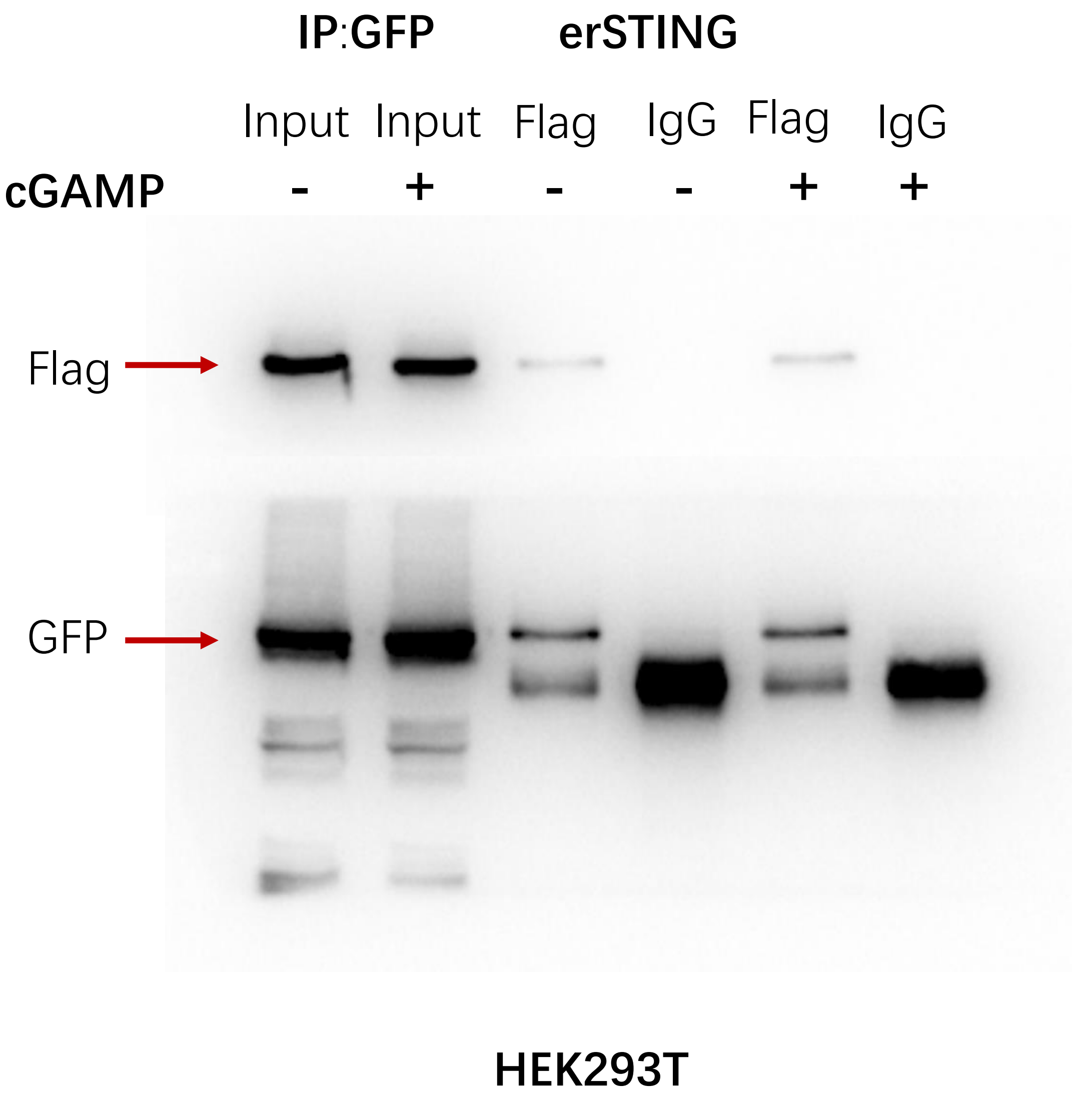

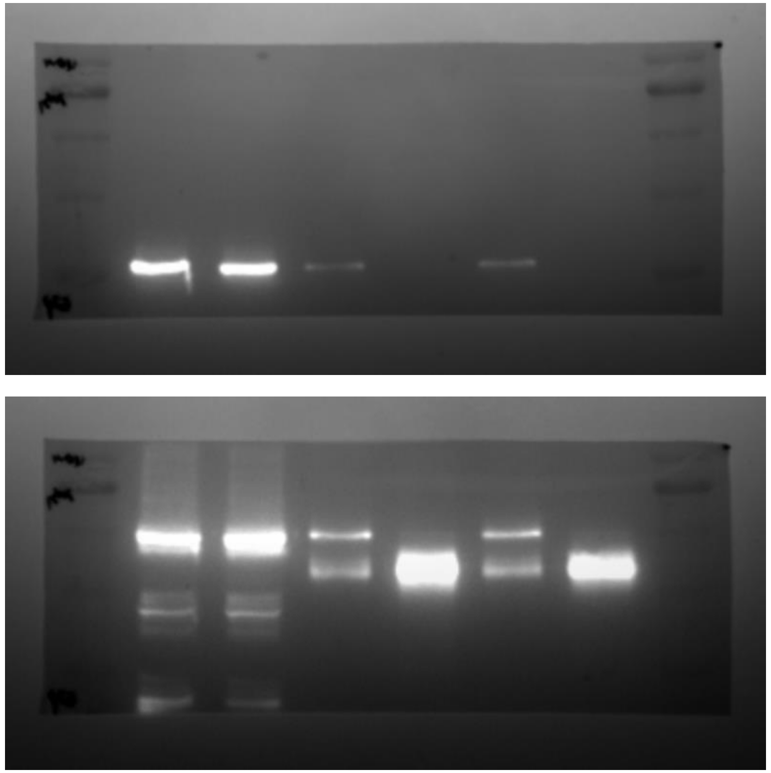

Full unedited gel for Figure 6

Figure 6D

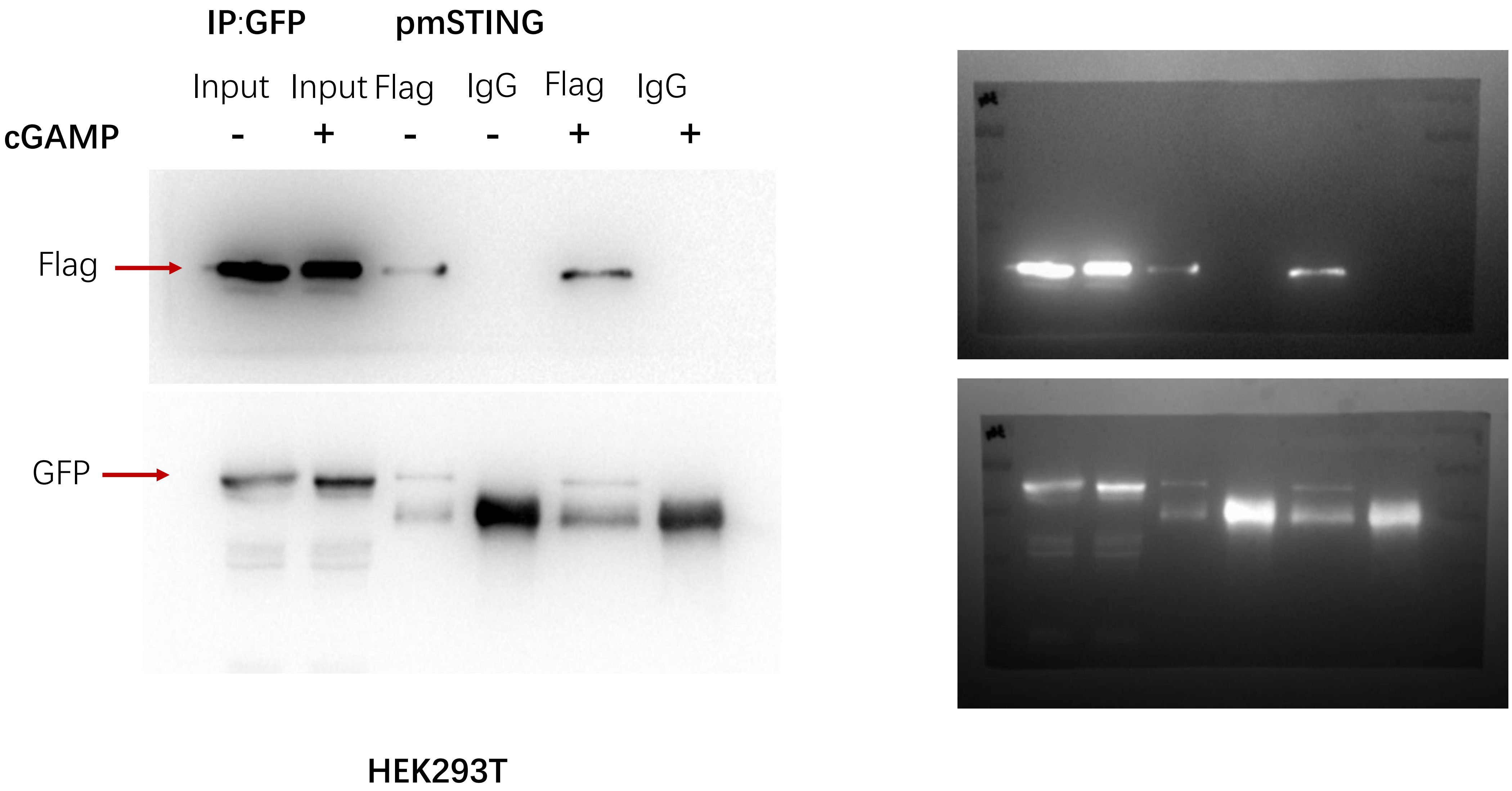

Full unedited gel for Figure 6

Figure 6E

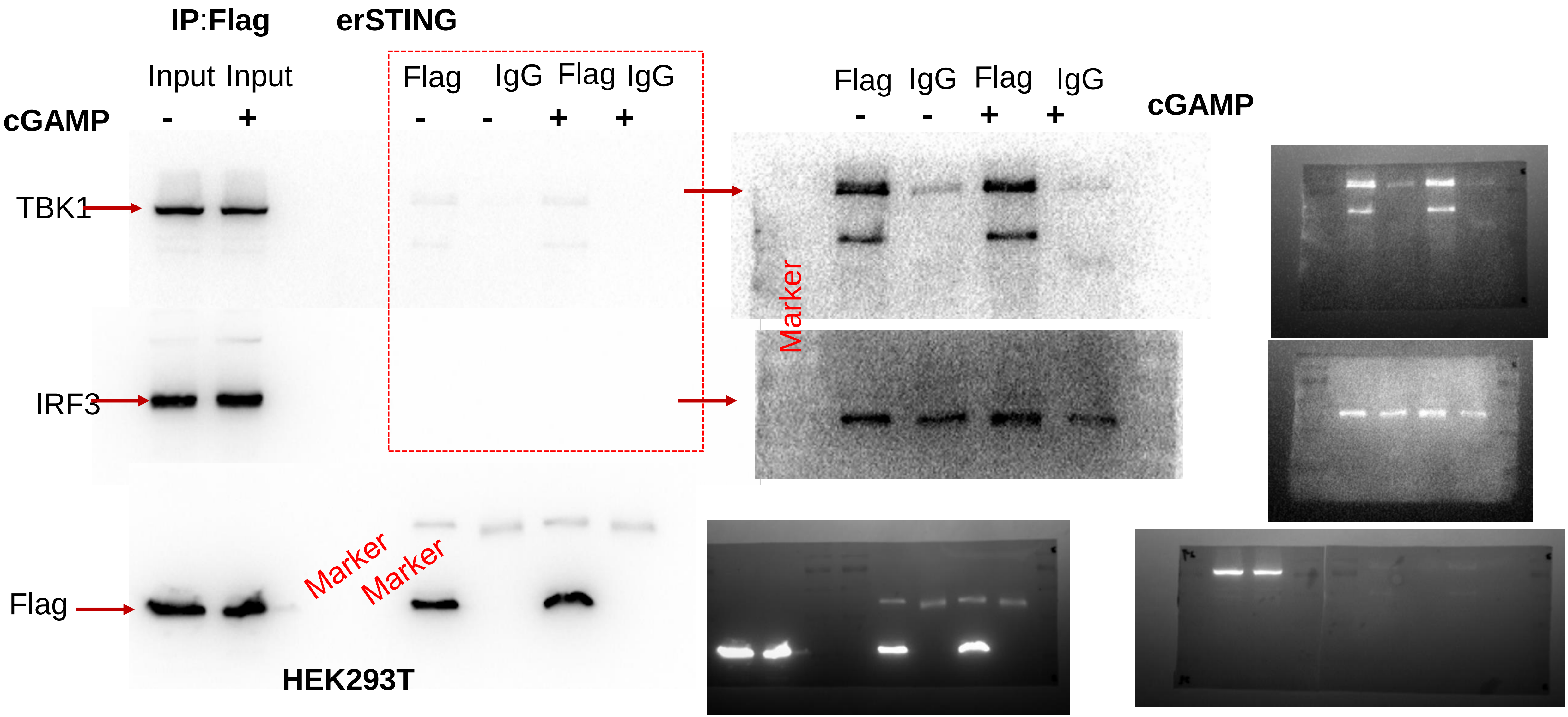

Full unedited gel for Figure 6

Figure 6E

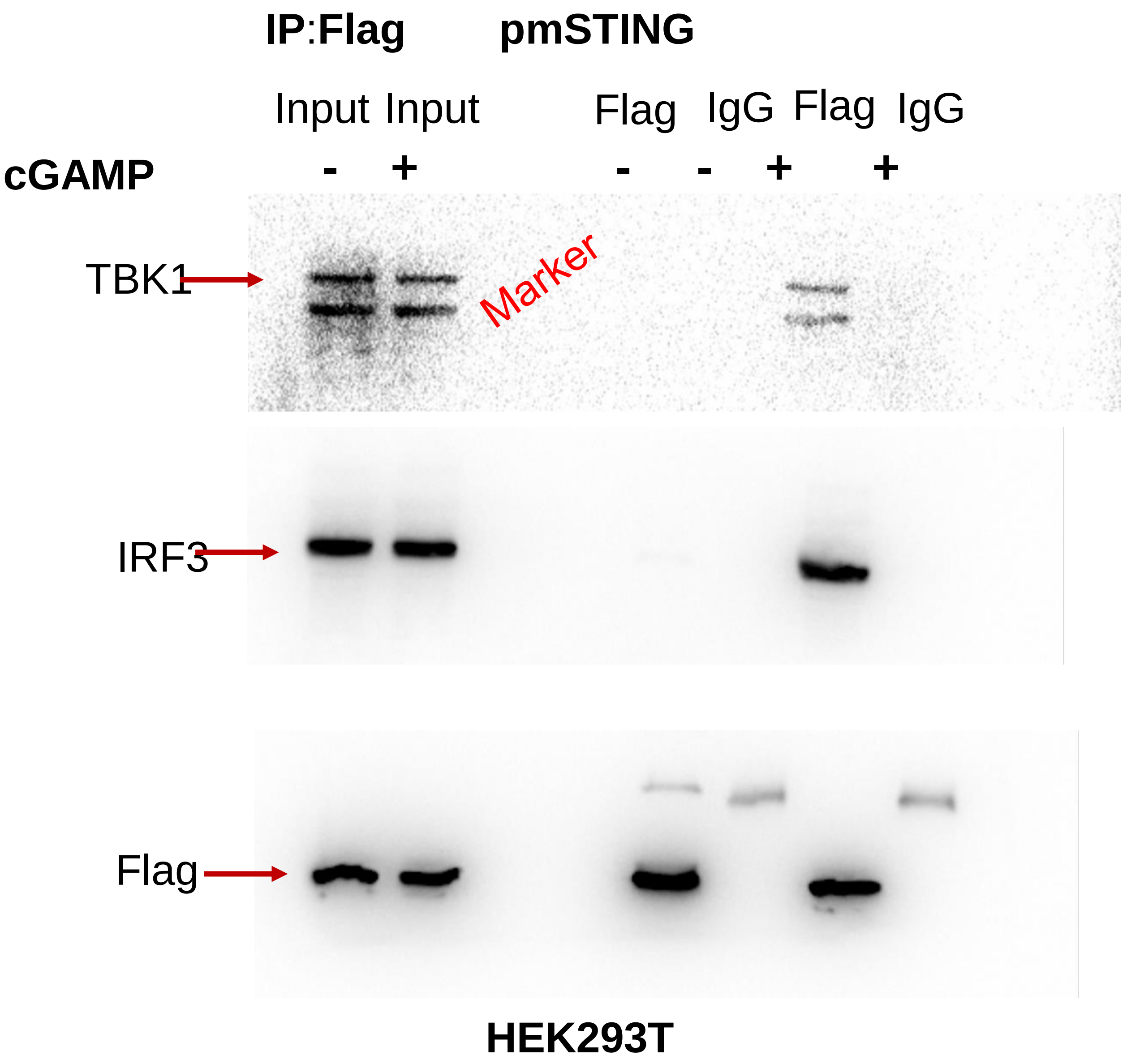

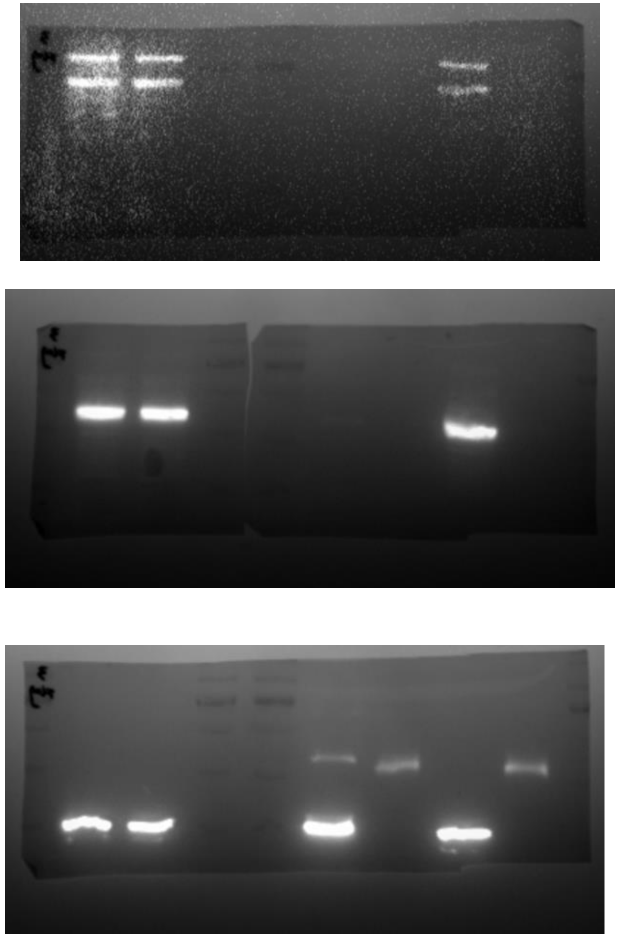

Full unedited gel for Figure S2

Figure S2 C

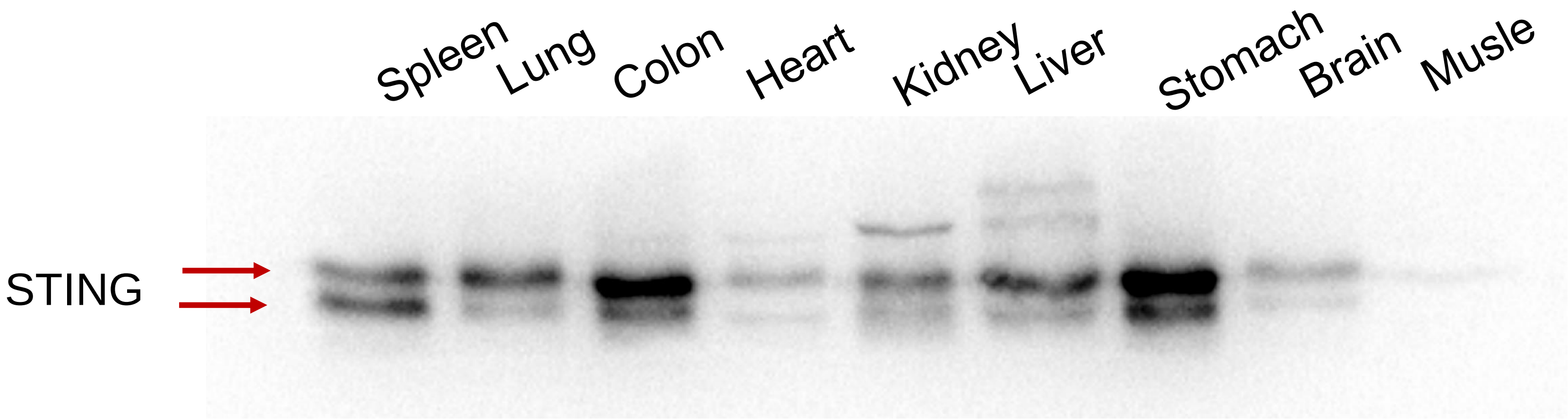

Figure S2 D

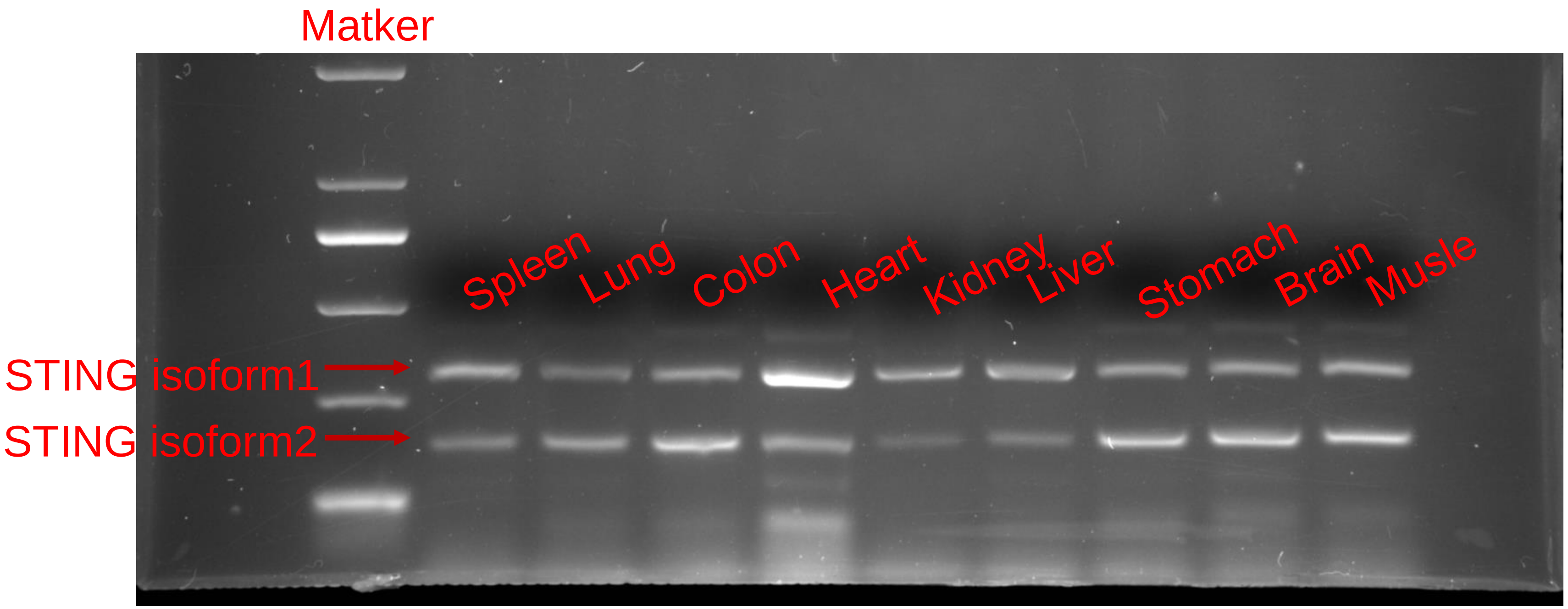

Full unedited gel for Figure S3

Figure S3 A

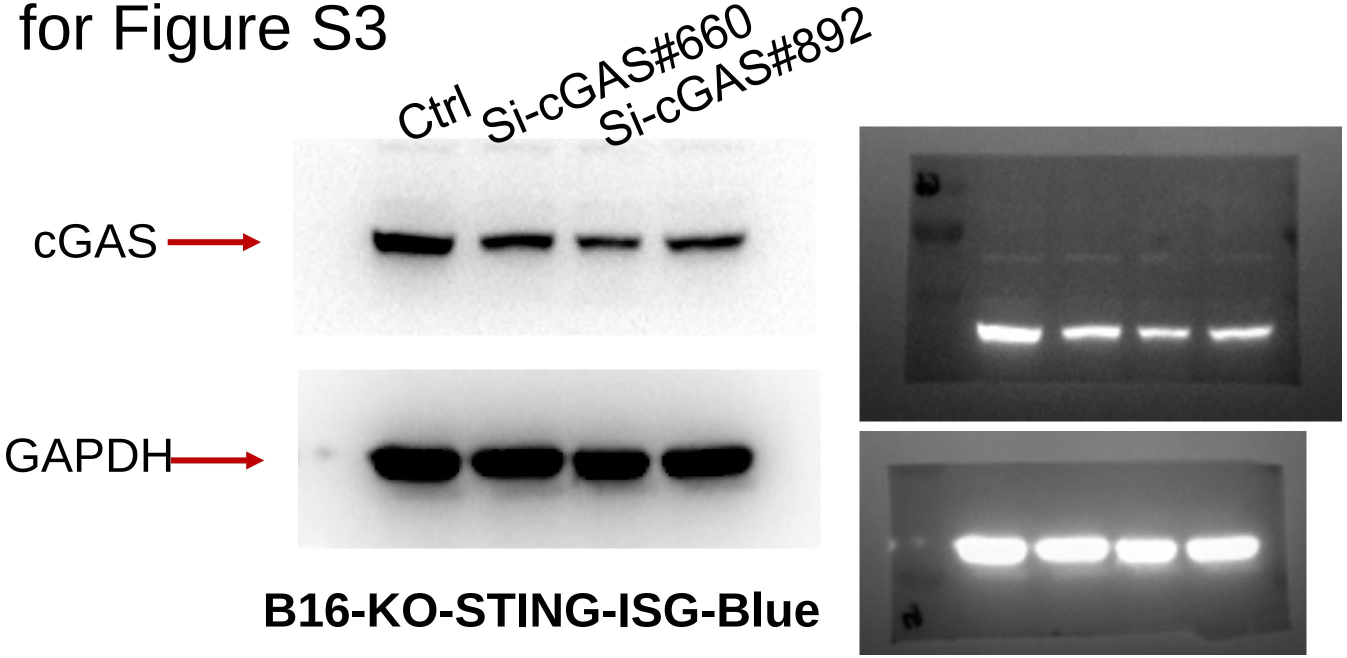

Figure S3 B

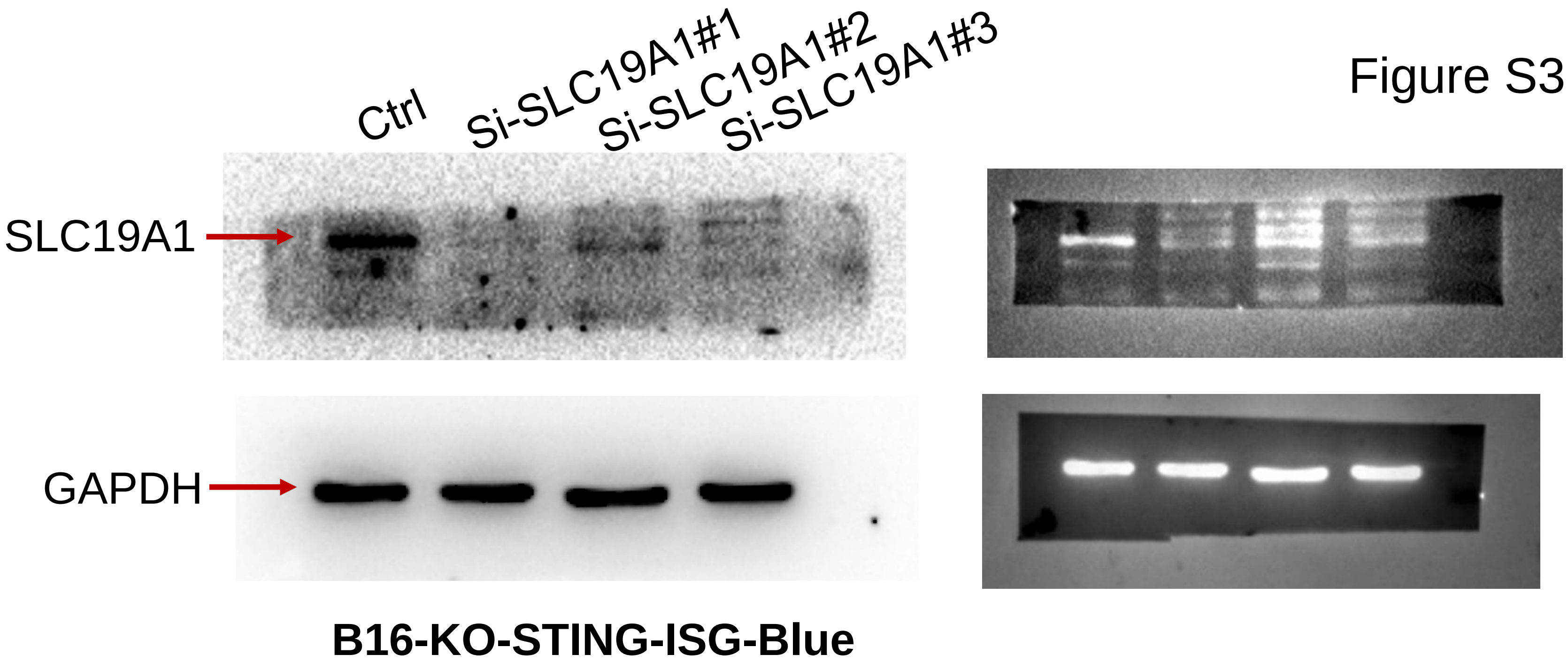

Supplement: Supplemental data [file jci-132-144339-s191.pdf]
